# A novel circular RNA, circIgfbp2, links neural plasticity and anxiety through targeting mitochondrial dysfunction and oxidative stress-induced synapse dysfunction after traumatic brain injury

**DOI:** 10.1038/s41380-022-01711-7

**Published:** 2022-08-02

**Authors:** Mengran Du, Chenrui Wu, Renqiang Yu, Yuqi Cheng, Zhaohua Tang, Biying Wu, Jiayuanyuan Fu, Weilin Tan, Qiang Zhou, Ziyu Zhu, Ehab Balawi, Xuekang Huang, Jun Ma, Z. B. Liao

**Affiliations:** 1 Department of Neurosurgery, The First Affiliated Hospital of Chongqing Medical University, Chongqing, 400016 China; 2 Department of Radiology, The First Affiliated Hospital of Chongqing Medical University, Chongqing, 400016 China

**Keywords:** Molecular biology, Neuroscience, Psychiatric disorders

## Abstract

Traumatic brain injury (TBI) can lead to different neurological and psychiatric disorders. Circular RNAs (circRNAs) are highly expressed in the nervous system and enriched in synapses; yet, the underlying role and mechanisms of circRNAs in neurological impairment and dysfunction are still not fully understood. In this study, we investigated the expression of circRNAs and their relation with neurological dysfunction after TBI. RNA-Seq was used to detect differentially expressed circRNAs in injured brain tissue, revealing that circIgfbp2 was significantly increased. Up-regulated hsa_circ_0058195, which was highly homologous to circIgfbp2, was further confirmed in the cerebral cortex specimens and serum samples of patients after TBI. Moreover, correlation analysis showed a positive correlation between hsa_circ_0058195 levels and the Self-Rating Anxiety Scale scores in these subjects. Furthermore, knockdown of circIgfbp2 in mice relieved anxiety-like behaviors and sleep disturbances induced by TBI. Knockdown of circIgfbp2 in H_2_O_2_ treated HT22 cells alleviated mitochondrial dysfunction, while its overexpression reversed the process. Mechanistically, we discovered that circIgfbp2 targets miR-370-3p to regulate BACH1, and down-regulating BACH1 alleviated mitochondrial dysfunction and oxidative stress-induced synapse dysfunction. In conclusion, inhibition of circIgfbp2 alleviated mitochondrial dysfunction and oxidative stress-induced synapse dysfunction after TBI through the miR-370-3p/BACH1/HO-1 axis. Thus, circIgfbp2 might be a novel therapeutic target for anxiety and sleep disorders after TBI.

## Introduction

Traumatic brain injury (TBI), associated with significant morbidity and mortality, leads to a tremendous economic burden on society and families [1]. Previous studies have suggested that TBI causes nerve damage and neurodegeneration, which may result in chronic neurocognitive, neurological, and psychological issues [2]. Recent studies have indicated that about 40% of patients with severe TBI develop psychiatric disorders, including depression, insomnia, anxiety, and sleep disorders, which can seriously affect the patients’ rehabilitation and quality of life [2, 3]. Nevertheless, the mechanisms leading to severe TBI are not fully elucidated. Also, clinical treatments for TBI and its complications are very limited. Thus, there is an urgent need to develop new treatment strategies for psychiatric disorders after TBI.

Mitochondrial dysfunction and oxidative stress are essential early pathological features of TBI [4, 5]. Previous studies have shown that mitochondrial oxidative stress leads to behavioral phenotypes, such as cognitive impairment and mood disorders [6]. In addition, it has been suggested that mitochondrial dysfunction in the cerebral cortex leads to disorders in the brain’s energy supply [7]. Mitochondrial dysfunction might occur due to alteration in synapse-related signal transduction [8] and might be involved in compromised synaptic development [9], thus forming a vicious circle. All these data imply that the inhibition of mitochondrial oxidative stress might have a crucial role in the recovery of the behavioral phenotype of TBI.

BTB and CNC homology 1 (BACH1) is a heme-binding transcription factor closely related to oxidative stress and inflammation in TBI. BACH1 inhibits the expression of the HMOX1 gene that encodes heme oxygenase-1 (HO-1) [10–12] and, along with its catalytic products, can protect tissues and cells through anti-oxidation and anti-inflammation effects [13, 14]. Nevertheless, the upstream factors regulating BACH1 remain unclear.

Circular RNA (circRNA) is a family of single-stranded circular RNAs [15, 16]. So far, many circRNAs that regulate gene expression at transcriptional, post-transcriptional, and translational levels have been identified. These circRNAs participate in many pathological processes such as Alzheimer’s disease, diabetes, atherosclerosis, and glioma by regulating alternative splicing, sponging miRNAs, sequestering functional proteins, or even encoding proteins [17–20]. Previous studies have found that the brain circRNA profiles were significantly altered after TBI in rats and mice [21]. A recent study conducted by our group also showed that melatonin reduced ferroptosis and improved sleep disorders via the circPtpn14/miR-351-5p/5-LOX after TBI [22]. Moreover, our previous study found that the circLphn3 protected the blood-brain barrier after TBI by binding miR-185-5p to up-regulate the tight junction protein ZO1 [23]. Some circRNAs, such as CDR1as [24], circRNA103636 [25], and circHomer1a [26], are involved in mood disorders. However, only a few studies have examined the association between circRNA and mood disorders after TBI.

In this study, we investigated whether circIgfbp2 was involved in post-traumatic mental and cognitive impairment. Next, the biological function of circIgfbp2 was analyzed in the TBI mouse model and H_2_O_2_-induced HT22 cells. Finally, the molecular mechanism of circIgfbp2 involving mitochondrial dysfunction and oxidative stress-induced synaptic dysfunction was demonstrated after TBI.

## Material and methods

### Ethical statement

The human study was conducted according to the declaration of Helsinki and approved by the ethics committee of the First Affiliated Hospital of Chongqing Medical University (No. 2019-215). Each patient or his/her legal representative signed written informed consent. All animal studies were done in compliance with the rules of Chongqing Medical University institutional animal care (No. 2021-177) and the National Institutes of Health Guidelines for the Care and Use of Laboratory Animals.

### Human brain specimens (tissue or blood) preparation

Contusion brain specimens (about 1 cm^3^) were obtained from 6 patients who underwent craniotomy within 5–20 h after severe TBI. Three control specimens were obtained in the surgical pathway from the cerebral cortex to the ventricles for intraventricular tumors (WHO I) (Supplementary Table 1). The contusion brain samples obtained during the operation were immediately frozen in liquid nitrogen and stored at −80 °C for subsequent tests.

A total of fifty patients with acute TBI were included in the analysis. Inclusion criteria were: (1) aged 16–55 years old; (2) recent head trauma with impaired consciousness; (3) initial pre-admission Glasgow Coma Scale (GCS) score 8–13; (4) the patient or his/her legal representative understood and signed the informed consent form. Exclusion criteria were the following: (1) previous history of psychiatric disorder, head trauma, or intracranial lesions; (2) pregnant women; (3) dropouts; (4) development of serious complications such as intracranial infection or multi-organ failure; (5) Patients hospitalized for longer than 30 days. The venous blood of fifty patients with acute TBI was drawn at admission, and the blood of 20 healthy people (volunteers) was used as a control sample. The details of patients and specimens are shown in Supplementary Table 2. The patients and investigators were not blinded to the treatment assignment. Blood samples were kept at −80 °C for subsequent tests.

### Anxiety-like and depression-like behavior tests in patients

The fifty patients with acute TBI were evaluated based on symptoms, signs, physical examination, radiological imaging, and GCS scores on admission and discharge. All patients were followed up by telephone once a month after discharge. The patients were evaluated for anxiety and depression by scales during the telephone follow-up 3 months after discharge. Anxiety-like behaviors were assessed using the Self-Rating Anxiety Scale (SAS). Depression-like behaviors were analyzed using the Self-Rating Depression Scale (SDS). The ROC curve analysis was performed by measuring serum hsa_circ_0058195 levels in the no anxiety TBI patients (SAS scores < 40) and the anxiety TBI patients (SAS scores > 40). Serum hsa_circ_0058195 levels were used as continuous values. The cut-off line for SAS score was set as 40 referencing a clinical study [27].

### Animals experiment

The animal sample size was determined using a sample size calculator (http://www.lasec.cuhk.edu.hk/sample-size-calculation.html). One hundred and eighty C57BL/6 male mice (aged 8–10 weeks and weighing 22–25 g) were obtained from the Animal Experimental Center of Chongqing Medical University. All the mice were housed in an environment with a temperature of 23 ± 1 °C, relative humidity of 50 ± 1%, and a light/dark cycle of 12/12 h. Mice were divided into six groups and were randomly assigned to groups using a simple randomization method (30 mice/group) as follows: sham, TBI, TBI with oe-circIgfbp2, TBI with oe-circ-NC, TBI with sh-circ-NC, and TBI with sh-circIgfbp2 groups. Fifteen mice in each group were used for behavioral tests, and the other mice in each group for the Western blotting, qRT-PCR, immunofluorescence staining, and ROS assay. Investigators were blinded to animal group assignments.

### Lentivirus injection into the left parietal lobe in mice

The circIgfbp2 (mmu_circ_0008937) overexpression lentivirus was synthesized by GeneSeed (Guangzhou, China), and an empty vector (oe-circ-NC) was used as a control. The circIgfbp2 knockdown lentivirus was synthesized by Hanbio (Shanghai, China), and an empty lentivirus was used as a control. CircIgfbp2 was knocked down using specific short interfering RNAs targeting the backsplice region. The sequences of sh-circIgfbp2 and sh-circ-NC were shown in Supplementary Table 3. The mice were anesthetized with 5% isoflurane and then mounted on a stereotaxic apparatus at the left parietal cortex with the following three injection coordinates: AP 1.0 mm, L 1.5 mm, H 1.0 mm; AP 1.0 mm, L 2.5 mm, H 1.0 mm; AP 1.5 mm, L 2.0 mm, H 1.0 mm (David Kopf Instruments, Tujunga, CA, USA). Then, the lentivirus (1 × 10^8^ IU per mouse) in 3 μL of PBS was injected (at 0.5 μL/min using a glass capillary) into the three allocated points in the left parietal cortex, which were used as the injury area for the controlled cortical impact (CCI) model 14 days later.

### TBI model

The CCI model was performed as previously described by our group [22]. The CCI model was induced by an electronically controlled pneumatic impact device (PSI, USA). After anesthesia induction with 5% isoflurane, the mouse head was fixed in the stereotactic frame. The CCI model striking parameters included the speed of 5.0 m/s, the depth of 1.5 mm, and the conduct of craniocerebral blow for 100 ms. In the sham group, only the skull was removed with no striking. And then, we **e**xamined the TBI model through the grasping test of the contralateral limb 2 h after TBI, and only successfully-modeled animals were included.

### Open field test

The open-field test was performed as previously described [28]. The open field was an uncapped Plexiglas box, with white light opaque squares on the inside and bottom, which were divided by black lines into 25 squares, with the central 9 cells being the central zone and the remaining cells being the peripheral zone. Initially, mice were placed in the center of the open field, and the percentage of mice remaining in the central zone within 5 min was recorded and calculated. The decreased percentage of time spent in the central zone suggested anxiety-like behavior.

### Tail suspension test

The tail suspension test was performed as previously described [28]. The mouse tail was fixed using adhesive tape, the tip of the nose was 25 cm from the tabletop, and the tail was pulled by a 4 cm long hollow hard, smooth plastic tube to stop the mouse from climbing the tail. The activity of the mice was observed within 5 min. The observation indexes included: resting time and resting latency.

### Elevated plus-maze test

The elevated plus-maze test was performed as previously described [28]. The device is a plus “+”-shaped, 50 cm elevated from the floor and divided into open arms, closed arms, and a center area. The arm’s length is 110 cm bilaterally, with no partitions on all sides of the open arms, while partitions surround the three peripheral sides of both of the closed arms, in which the external environment could not be seen. Mice were brought into the open field arena in the laboratory 30 min in advance before the formal test started. The mice were placed into the central area at the start of the experiment with the head facing one of the open arms.

### Electroencephalogram (EEG)

The EEG examination was performed as previously described by our group [22]. The EEG was performed on the 30th day after TBI. Before monitoring, mice were intraperitoneally injected with 3.5% chloral hydrate (Macklin, Cat#C804539, China, 5 ml/kg) to induce sleep (Natus Medical, USA). Two active electrodes were implanted in the scalp of the ipsilateral and contralateral parietal cortex, and two reference electrodes were implanted in the mastoid process. Sleep EEG waves of each mouse were analyzed by an EEG viewer (Natus Neurology Inc., Middleton, Wisconsin, USA) (α, β, θ, and δ waves).

### RNA-seq and bioinformatics analysis

The brain tissue specimens were obtained and snap-frozen in liquid nitrogen for transcriptome sequencing (BGI, China) after the mice were euthanized using CO_2_. The raw sequencing data were called raw tags. The raw tags were pruned using the following criteria for removal, in the following order: low-quality tags; tags with five primer contaminants; tags without three primers; tags without insertion; tags with poly-A; tags shorter than 18 nt. After filtering, the clean tags were mapped to the reference genome and other RNA databases, including miRbase, siRNA, piRNA, and snoRNA with Bowtie2. Particularly, cmsearch was performed for Rfam mapping. The software miRDeep2 was used to predict novel miRNA by exploring the secondary structure. RNAhybrid was used to predict the target genes of the miRNAs. The RNA expression level was calculated by counting the absolute numbers of molecules using unique molecular identifiers. Differential expression analysis was performed using the DEGseq, using Q-value ≤ 0.01 and the |fold change|å 2 as the default threshold to judge the significance of expression differences. The length, splicing mode, and sequence of circRNAs were obtained by circBase [29] and the UCSC genome browser. CircRNA-specific splice junction sequences were obtained by Sanger sequencing (BGI, China).

### Cell culture and transient transfection

HT22 cells and 293 T cells were purchased from OTWO BIOTECH (Shenzhen, China) and have been authenticated recently. HT22 cells were cultured in low glucose DMEM containing 10% fetal bovine serum in a humidified atmosphere containing 5% CO_2_ at 37 °C, 293 T cells were cultured in high glucose DMEM containing 10% fetal bovine serum in a humidified atmosphere containing 5% CO_2_ at 37 °C. All miRNAs mimics and anti-miRNAs were purchased from RiboBio (Guangzhou, China). HT22 cells were transfected with the lentivirus or the plasmids using Lipofectamine^TM^ 2000 (Life Technologies, Carlsbad, CA, USA), according to the manufacturer’s instructions. After lentivirus transfection for 7 days or liposome transfection for 2 days, the efficiency of transfection was determined by qRT-PCR. The concentrations of H_2_O_2_ in the culture medium were 600 μM/L. Wild-type and mutant psiCHECK2-circIgfbp2 plasmids and Wild-type and mutant psiCHECK2-BACH1 mRNA 3’UTR plasmids for the dual-luciferase reporter system were purchased from Tsingke Biotechnology Co. (Beijing, China).

### Isolation of mitochondria

The isolation of mitochondria from brain specimens or HT22 cells was performed according to a manufacturer’s protocol (Nanjing Jiancheng, Nanjing, China). ATP of mitochondria was measured according to the manufacturer’s protocol (Nanjing Jiancheng, Nanjing, China).

### Intracellular ROS assay

In order to measure the mitochondrial ROS levels in HT22 cells, the MitoSOX^TM^ Red Mitochondrial Superoxide Indicator Kit (Yeasen, China) was used. The MitoSOX^TM^ Red reagent working stock solution (5 μM) was added to the cells. The cells were incubated for 10 min at 37 °C in the dark, after which they were washed and resuspended in 500 μl PBS. The ROS levels were examined under a laser-scanning confocal microscope (Zeiss LSM800, Germany). The mitochondrial ROS levels of the brain tissues were measured according to the manufacturer’s protocol (Nanjing Jiancheng, Nanjing, China).

### RNA preparation and quantitative real-time PCR (qRT-PCR)

RNA Extraction Kit (Bio-Tek, Winooski, VT, USA) was used to extract total RNA, which was mixed with the reaction solution conFig.d with reverse transcription reagent (RT Master Mix for qPCR Kit, MedChemExpress, Monmouth Junction, NJ, USA), and placed into gradient PCR instrument for reverse transcription reaction. RNase R treatment was performed at 37 °C for 15 min using 3 U/mg RNase R (Epicentre, Madison, WI, USA). The cDNA formed by reverse transcription was then conFig.d with SYBR green (SYBR^®^ Green qPCR Master Mix, MedChemExpress, Monmouth Junction, NJ, USA). The primers for mmu-miR-370-3p were purchased from RiboBio (China), and the sequence of mmu-miR-370-3p was 5ʹ-GCCUGCUGGGGUGGAACCUGGU-3ʹ. The sequences of the primers for the detection of circIgfbp2, Igfbp2, BACH1, and GAPDH are shown in Supplementary Table 4.

### Double-labeled immunofluorescence

The prepared frozen brain sections and HT22 cells were incubated with the appropriate antibodies (two mixed antibodies) overnight at 4 °C and then incubated with a secondary antibody conjugated with Fluor 488/594 (Invitrogen) for 1 h at room temperature. A laser-scanning confocal microscope (Zeiss LSM800, Germany) and ImageJ software were then used to examine photomicrographs. The antibodies were HO-1 antibody (ZEN BIO, 384541, China), PSD95 (GeneTex, GTX22723, USA), and Syn (GeneTex, GTX100865, USA).

The fluorescence in situ hybridization (FISH) kit was obtained from RiboBio (China), and FISH probes for circIgfbp2 and miR-370-3p were purchased from GeneSeed (China). FISH was performed according to the protocol of the kit.

### Western blot

The extracted brain tissues or cells were added into RIPA lysate and then fully ground. After centrifugation, the supernatant was aspirated. After the BCA protein quantitative kit determined the protein concentration, each sample was balanced with RIPA lysis solution. The protein samples were stored at −20 °C after boiling and denaturing. An equal amount of protein sample was added to each loading well of SDS gel for electrophoresis. According to the molecular weight, the corresponding position of SDS gel was cut off and transferred to the PVDF membrane. After incubating the PVDF bands in the blocking solution, the PVDF bands were placed in the corresponding first anti-antibody (1:1000) and incubated overnight at 4 °C. The PVDF bands were added to the HRP-conjugated secondary antibody (1:8000) and incubated for 1 h. Finally, ECL chemiluminescent developer was used to develop and photograph the bands, and ImageJ analysis software was used to calculate the relative expression of proteins. β-actin (GeneTex, GTX109639, USA) was used as an internal reference. Other major antibodies have been mentioned above.

### Dual-luciferase reporter system

In the psiCHECK2 plasmid (HanBio, Shanghai, China), the target circIgfbp2 sequence or the target BACH1 mRNA 3’UTR sequence was cloned downstream of the hRluc gene to construct two reporter vectors containing the potential binding site of miR-370-3p. Next, two different reporter vectors and miR-370-3p mimics were co-transfected into 293 T cells, and Renilla luciferase activity and firefly luciferase activity were determined by Dual-Lumi™ II Luciferase Reporter Gene Assay Kit (BeyoTime, China). Relative luciferase activity = Renilla luciferase activity/firefly luciferase activity.

### Pull-down assay with biotinylated circIgfbp2 and miR-370-3p

The biotinylated probe of circIgfbp2 back splice sequence and miR-370-3p were designed and synthesized by RiboBio (China). HT22 cells were transfected with biotinylated probes fusing Lipofectamine RNAiMax (Life Technologies, Gaithersburg, Maryland, USA). 48 h after the transfection, the cells were collected, washed with PBS, and incubated with lysis buffer (Ambion, Austin, TX, USA) on ice for 10 min. Then, 10% of the cell lysates were input. To generate probe-coated magnetic beads, the biotinylated probe was resuspended in washing/binding buffer (0.5 mol/L NaCl, 20 mmol/L Tris HCl, pH 7.5, and 1 mmol/L EDTA) and then incubated with Dynabeads MyOne Streptavidin C1 (Thermo Fisher) for 4 h at 4 °C. Subsequently, HT22 cells were lysed, sonicated, and cultured overnight with the target probe or control probe at 4 °C. After treatment with washing/binding buffer and RNeasy Mini Kit (Qiagen, Venlo, the Netherlands), RNA complexes bound to beads were isolated according to standard procedures for further qRT-PCR analysis.

### Statistical analyses

Statistical analysis and plotting were performed using GraphPad Prism 8 (GraphPad Software Inc., San Diego, CA, USA). All data are presented as the mean ± SEM. Two independent groups were analyzed with a two-tailed t-test. Sets of data (>2 groups) were analyzed using one-way, or two-way ANOVA followed by Tukey’s multiple comparisons test. And we checked the homoscedasticity between groups being statistically tested using Bartlett’s test. *P*-value < 0.05 was considered statistically significant.

## Results

### CircIgfbp2 is significantly up-regulated after TBI in mice and in H_2_O_2_-treated HT22 cells

**Fig. 1:**
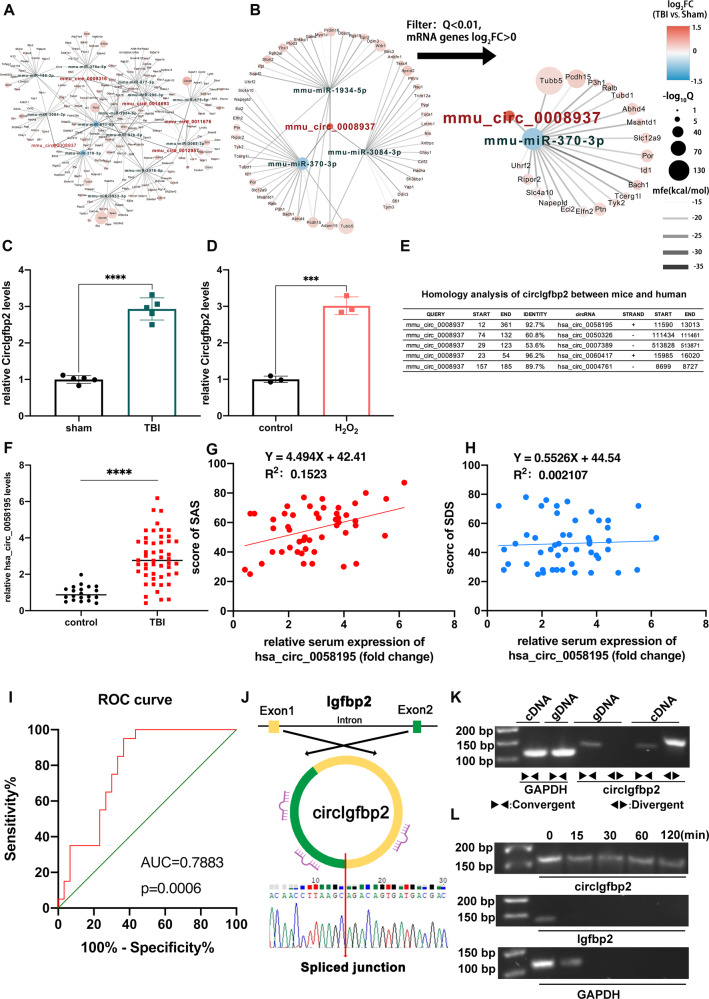
circIgfbp2 is up-regulated in the TBI mice and H_2_O_2_-treated cells. Also, hsa_circ_0058195 is elevated in acute TBI patients and is involved in anxiety-like behaviors after TBI. **A**, **B** The network map of the differential circRNAs-miRNAs-mRNAs genes regulatory network screening by DEG algorithm, showing the top 5 up-regulated circRNAs (sham group vs. TBI group) (|fold-change| > 2, Q < 0.01). Red: circRNAs; Green: miRNAs; Black: mRNAs. **C** Relative circIgfbp2 levels were measured by qRT-PCR in the sham and injured brain tissue 3 days after TBI. *n* = 5 mice per group. ****p < 0.0001, two-tailed t-test. **D** Relative circIgfbp2 levels in the H_2_O_2_-treated HT22 cells and control group. *n* = 3 replications, ***p < 0.001, two-tailed t-test. **E** Homology analysis between mice circIgfbp2 and human circRNAs. **F** The relative expression level of hsa_circ_0058195 in the serum of acute TBI patients, which was highly homologous to circIgfbp2, and up-regulated after TBI. *n* = 50 TBI patients, *n* = 20 volunteers as control, ****p < 0.0001, two-tailed t-test. **G** The anxiety-like behaviors were analyzed using the Self-Rating Anxiety Scale in patients 3 months after TBI. The scale score had a positive linear relationship with the expression of hsa_circ_0058195. *n* = 50 TBI patients, *n* = 20 volunteers as control, *p* = 0.0051. **H** The depression-like behaviors were analyzed using the Self-Rating Depression Scale in patients 3 months after TBI. The scale score had no linear relationship with the expression of hsa_circ_0058195. *n* = 50 TBI patients, *n* = 20 volunteers as control, *p* = 0.7519. **I** ROC curve in the evaluation of the diagnostic value of serum hsa_circ_0058195 for anxiety after TBI. Area under curve (AUC) = 0.7883; *p* = 0.0006. **J** Schematic representation of the circularization of Igfbp2 1-2 exons to form circIgfbp2. The results of Sanger sequencing of the spliced junction resulting from the divergent primers are shown. **K** The presence of circIgfbp2 was validated in HT22 cells by PCR and agarose gel electrophoresis (AGE). Divergent primers amplified circIgfbp2 from cDNA but not from gDNA. **L** CircIgfbp2, linear Igfbp, and GAPDH levels were detected by PCR and AGE treated with RNase R for 0 to 120 min. All data were represented as mean ± SEM.

The expression profiles of circRNAs are changed after TBI [21, 22], but the involvement of specific circRNAs in neuropsychological impairment after TBI remains unclear. In order to examine the differentially expressed circRNAs after TBI, we collected three pairs of sham and traumatic mouse brain specimens and conducted the RNA-Seq (PRJNA725662). Results revealed 640 upregulated and 1049 downregulated circRNAs (all with |fold-change|>2, Q < 0.01). From the differentially expressed circRNAs after TBI, we screened five up-regulated circRNAs with the largest fold change. In addition, according to the sequencing results and RNAhybrid algorithm, we mapped a circRNAs-miRNAs-mRNAs interaction network after TBI in mice (Fig. 1A). The RNAhybrid algorithm was used to predict potential miRNAs binding sites of circRNAs (minimum free energy < −25 kcal/mol). We found that circIgfbp2 had the most predicted binding miRNAs among the five up-regulated circRNAs, which may be more powerful in regulating the translation. Therefore, circIgfbp2 was chosen as the target for our following experiments (Fig. 1B).

Next, we verified the expression of circIgfbp2 in vivo and in vitro. circIgfbp2 was significantly up-regulated in mice’s damaged brain tissues (*P* < 0.0001, Fig. 1C) and H_2_O_2_-treated HT22 cells (*P* < 0.001, Fig. 1D), suggesting that circIgfbp2 may have an essential regulatory role in TBI.

### Hsa_circ_0058195 is up-regulated in the cerebral cortex tissue and serum of TBI patients with post-traumatic anxiety

Some circRNAs have been associated with mood disorders [24–26], but none were explicitly examined in relation to mood disorders after TBI. The host genes of mmu_circ_0008937 (circIgfbp2) and hsa_circ_0058195 are *Igfbp2* and *IGFBP2*, respectively, which are homologous genes. In addition, a comparison of the sequences of mmu_circ_0008937 with the human circRNAs database by BLAST revealed hsa_circ_0058195 with 92.7% identity with mmu_circ_0008937 (Fig. 1E). In this study, we found higher levels of hsa_circ_0058195 in the serum of TBI patients compared to healthy volunteers (*P* < 0.0001, Fig. 1F). Then, the SAS and SDS were used to determine the mental condition, revealing that the hsa_circ_0058195 expression level was positively correlated with the SAS scores (Fig. 1G) but not with the SDS scores (Fig. 1H). In addition, the receiver operating characteristic (ROC) curve analysis suggested that the hsa_circ_0058195 levels could be used to diagnose anxiety after TBI in patients (*P* < 0.001, Fig. 1I). These results imply that increased circIgfbp2 expression levels might be associated with anxiety induced by TBI.

### CircIgfbp2 has an essential role in regulating anxiety-like behaviors in mice after TBI

CircIgfbp2 has a spliced sequence length of 368 bp and is formed by exons 1–2 of Igfbp2 (Fig. 1J). qRT-PCR analysis showed that the circIgfbp2 could be amplified by divergent primers in complementary DNA (cDNA) but not in genomic DNA (gDNA), while the linear Igfbp2 could be amplified by convergent primers from cDNA and gDNA (Fig. 1K). In addition, the stability of circIgfbp2 was significantly higher than linear Igfbp2 under RNase R intervention (Fig. 1L).

**Fig. 2:**
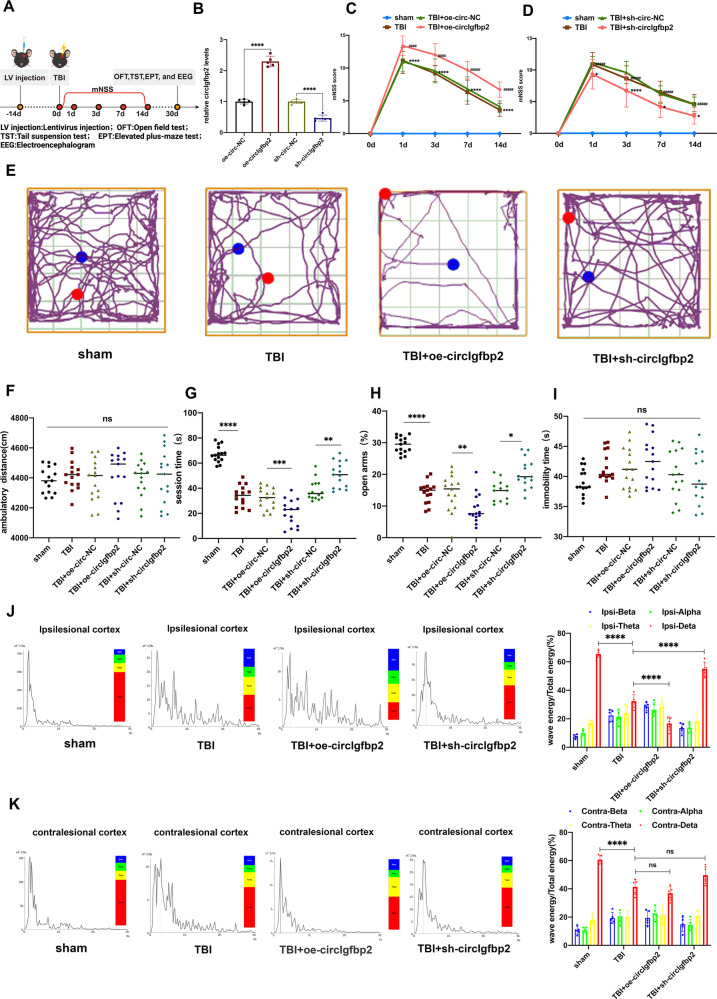
circIgfbp2 has an important role in regulating anxiety-like behaviors and sleep disorders in mice after TBI. **A** Illustration of the experimental procedure. **B** The relative expression level of circIgfbp2 after injection of lentivirus carrying an overexpression or inhibition circIgfbp2 at the left parietal lobe in mice for 14 days (*n* = 5 mice per group). **C** Neurological deficits were measured by calculating the modified Neurological Severity Scores (mNSS) at 0, 1, 3, 7, and 14 days after TBI. oe-circIgfbp2: circIgfbp2 overexpression. *n* = 15 per group. TBI + oe-circIgfbp2 vs. TBI + oe-circ-NC, ^####^*p* < 0.0001. TBI vs. the sham, ****p < 0.0001, two-way repeated-measures ANOVA followed by Tukey’s multiple comparisons test. **D** mNSS was measured after circIgfbp2 knockdown. sh-circIgfbp2: circIgfbp2 knockdown. *n* = 15 mice per group. TBI + sh-circIgfbp2 vs. TBI + sh-circ-NC group, at 1 day, *p < 0.05; at 3 days, ****p < 0.0001; at 7 and 14 days, *p < 0.05. TBI vs. sham, ^####^*p* < 0.0001, two-way repeated-measures ANOVA followed by Tukey’s multiple comparisons test. **E**–**G** The anxiety-like behaviors were measured using the open field test 30 days after TBI. The representative trails of the open field test were presented, *n* = 15 mice per group. Distance (**F**): *p* = 0.9578, ns. Central area movement time (**G**): TBI vs. sham, ****p < 0.0001; TBI + oe-circIgfbp2 vs. TBI + oe-circ-NC, ***p < 0.001; TBI + sh-circIgfbp2 vs. TBI + sh-circ-NC, **p < 0.01, one-way repeated measures ANOVA followed by Tukey’s multiple comparisons test. **H** The anxiety-like behaviors were measured by an elevated plus-maze test 30 days after TBI, *n* = 15. *P* < 0.0001. TBI vs. sham, ****p < 0.0001; TBI + oe-circIgfbp2 vs. TBI + oe-circ-NC, **p < 0.01; TBI + sh-circIgfbp2 vs. TBI + sh-circ-NC, *p < 0.05, one-way repeated measures ANOVA followed by Tukey’s multiple comparisons test. **I** The depression-like behavior was measured by the tail suspension test 30 days after TBI, *n* = 15 per group. *P* = 0.1050, ns. One-way repeated-measures ANOVA followed by Tukey’s multiple comparisons test. **J**, **K** Representative energy spectral density charts and waveforms in the ipsilesional and contralesional cortex in different groups 30 days after TBI, measured by EEG. Percentage of each wave energy (Red: delta; Yellow: theta; Green: alpha; and Blue: beta) relative to the total energy in contralesional and ipsilesional cortices in different groups. *n* = 5 mice per group. In ipsilesional cortex groups, for delta: TBI vs. sham, ****p < 0.0001;TBI + oe-circIgfbp2 vs. TBI, ****p < 0.0001; TBI + sh-circIgfbp2 vs. TBI, ****p < 0.0001. In contralesional cortex groups, for delta: TBI vs. sham, ****p < 0.0001; TBI + oe-circIgfbp2 vs. TBI, *p* > 0.05, ns; TBI + sh-circIgfbp2 vs. TBI, *p* > 0.05, ns. One-way ANOVA followed by Tukey’s multiple comparisons test. ns: no significance. All data were represented as mean ± SEM.

In order to study whether circIgfbp2 could regulate mood disorders induced by TBI, we knocked down and overexpressed circIgfbp2 in the left parietal lobe cortex of the mice through lentivirus injection 14 days before TBI (Fig. 2A). The knockdown and overexpression efficiencies of circIgfbp2 were determined by qRT-PCR after lentivirus injection for 14 days. The results confirmed the efficiency of the overexpression and knockdown circIgfbp2 with lentiviruses (*P* < 0.0001, Fig. 2B). In addition, neurological deficits were assessed using the well-established modified neurological severity score (mNSS). The mNSS score was 0 before TBI; it peaked one day after TBI and then progressively decreased over time in all groups, and the overexpression of circIgfbp2 significantly increased the mNSS score at all time points after TBI (*P* < 0.0001, Fig. 2C), while the knockdown of circIgfbp2 significantly decreased the mNSS score at all time points after TBI (*P* < 0.05, Fig. 2D), which suggested that knockdown of circIgfbp2 could promote the neurological recovery.

The results of the open field tests (Fig. 2E) showed that there was no significant difference in the total movement distance of TBI mice that knocked down circIgfbp2 compared with TBI mice. (*P* > 0.05, Fig. 2F), indicating that the inhibition of circIgfbp2 did not affect the mice’s motor ability. Interestingly, the knockdown of circIgfbp2 increased exploration behavior in the central area (*P* < 0.01, Fig. 2G), suggesting that inhibiting the circIgfbp2 could improve anxiety after TBI. Meanwhile, the elevated plus-maze test showed that knockdown of circIgfbp2 increased the time of opening arms (*P* < 0.05, Fig. 2H), while knockdown of circIgfbp2 reduced the TBI-induced anxiety-like behaviors. On the other hand, the suspension tail test showed no difference among these groups (*P* > 0.05, Fig. 2I), revealing that circIgfbp2 had no significance in depressive behavior.

Moreover, sleep EEG revealed that the wave’s energy spectral density in the ipsilesional cortex of TBI mice was impaired compared with the sham group. Surprisingly, the energy of the delta waves in the ipsilesional cortex was significantly elevated in the TBI + sh-circIgfbp2 group compared with the TBI group while significantly decreased in the TBI + oe-circIgfbp2 group (*P* < 0.0001, Fig. 2J). Furthermore, the energy of the delta waves in the contralesional cortex showed no differences in the TBI + sh-circIgfbp2 group compared with the TBI group and TBI + oe-circIgfbp2 group (*P* > 0.05, Fig. 2K). To sum up, EEG reflects the regulatory effect of circIgfbp2 on sleep disorders after TBI, and the results suggest that circIgfbp2 knockdown alleviates sleep disturbances after TBI.

Overall, the above results indicate that the knockdown of circIgfbp2 relieves anxiety-like behaviors and sleep disturbances induced by TBI in vivo.

### Knockdown of circIgfbp2 regulates brain damage and mitochondrial dysfunction in TBI mice

**Fig. 3:**
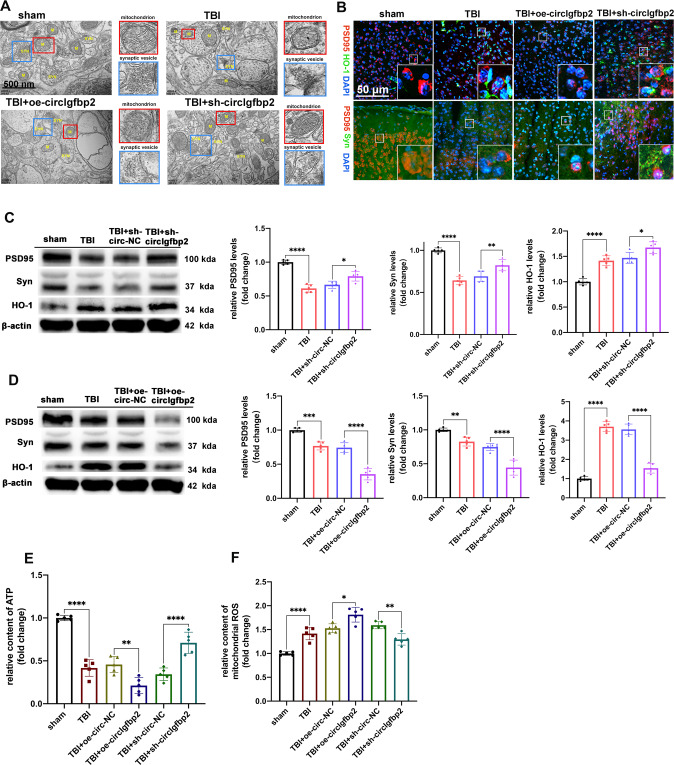
Knockdown of circIgfbp2 alleviates brain injury after TBI, while overexpression of circIgfbp2 reverses this process. **A** The effect of circIgfbp2 overexpression or knockdown on the brain ultrastructure three days after TBI. TEM photomicrographs showed the changes in mitochondria and synaptic vesicles in the different groups. The red boxed regions indicated mitochondria, while the blue boxed regions indicated synaptic vesicles. **B** The co-localization of PSD95 with HO-1 or Syn was detected by double immunofluorescence in sham, TBI, TBI + oe-circIgfbp2, TBI + sh-circIgfbp2 group. **C** The expression of PSD95, Syn and HO-1 after circIgfbp2 knockdown in mice at 3 days after TBI by Western blot. *n* = 5 per groups, PSD95: TBI vs. sham, ****p < 0.005, TBI + sh-circIgfbp2 vs. TBI + sh-circ-NC, *p < 0.05; Syn: TBI vs. sham, ****p < 0.0001, TBI + sh-circIgfbp2 vs. TBI + sh-circ-NC, **p < 0.01; HO-1: TBI vs. sham, ****p < 0.0001, TBI + sh-circIgfbp2 vs. TBI + sh-circ-NC, *p < 0.05. One-way ANOVA followed by Tukey’s multiple comparisons test. **D** The expression of PSD95, Syn and HO-1 after circIgfbp2 overexpression in TBI mice at 3 days by Western blot. *n* = 5 mice per group, PSD95: TBI vs. sham, ***p < 0.001, TBI + oe-circIgfbp2 vs. TBI + oe-circ-NC, ****p < 0.0001; Syn: TBI vs. sham, **p < 0.001, TBI + oe-circIgfbp2 vs. TBI + oe-circ-NC, ****p < 0.0001; HO-1: TBI vs. sham, ****p < 0.0001, TBI + oe-circIgfbp2 vs. TBI + oe-circ-NC, ****p < 0.0001. One-way ANOVA followed by Tukey’s multiple comparisons test. **E** The content of ATP of circIgfbp2 overexpression or knockdown mice after TBI, *n* = 5 mice per group. TBI vs. sham, ****p < 0.0001, TBI + oe-circIgfbp2 vs. TBI + oe-circ-NC, **p < 0.01,TBI + sh-circIgfbp2 vs. TBI + sh-circ-NC, ****p < 0.0001.One-way ANOVA followed by Tukey’s multiple comparisons test. **F** The content of mitochondrial ROS of circIgfbp2 overexpression or knockdown in mice at 3 days after TBI, *n* = 5 mice per group. TBI vs. sham, ****p < 0.0001, TBI + oe-circIgfbp2 vs. TBI + oe-circ-NC, *p < 0.05, TBI + sh-circIgfbp2 vs. TBI + sh-circ-NC, **p < 0.01.One-way ANOVA followed by Tukey’s multiple comparisons test. All data were represented as mean ± SEM.

TBI is characterized by brain ultrastructural changes, which are markers of the extent of brain damage [30]. Transmission electron microscopy was performed to observe the ultrastructural changes after TBI. Abnormal mitochondrial membrane swelling and rupture of synaptic vesicles (which are characteristic ultrastructural changes after TBI [31]) were aggravated by the overexpression of circIgfbp2, while knockdown of circIgfbp2 reversed this process after TBI (Fig. 3A). Therefore, inhibiting circIgfbp2 can reduce the ultrastructural damage after TBI.

As indicated above, circIgfbp2 knockdown alleviates brain damage and improves anxiety after TBI; yet, the molecular mechanism remains unknown. TBI, which has a significant impact on synapse structure and function required for synaptic plasticity and cognitive function, often leads to synapse loss [32]. PSD95 is a scaffold protein involved in synapse plasticity [33], Syn is involved in neurotransmission [34], and HO-1 has a protective effect on neurons by decreasing oxidative stress [13]. In this study, we found that knockdown of circIgfbp2 increased HO-1 and alleviated the loss of PSD95 and Syn after TBI (Fig. 3B, C), while overexpression of circIgfbp2 aggravated those changes (Fig. 3D). Meanwhile, knockdown of circIgfbp2 increased the mitochondrial ATP content and decreased the content of mitochondrial ROS, while the results of overexpression of circIgfbp2 showed that mitochondrial oxidative stress was aggravated (*P* < 0.05, Fig. 3E, F).

These results indicate that circIgfbp2 knockdown reduces neuron damage and improves neuronal synaptic plasticity by decreasing oxidative stress and mitochondrial damage after TBI.

### CircIgfbp2 targets miR-370-3p to regulate BACH1

We illustrated the screening of candidate circRNAs and the suggested possible roles of circIgfbp2 in brain injury (Figs. 1–3). Further experiments were performed to analyze the mechanisms. qRT-PCR results showed that the circIgfbp2 overexpression lentivirus and the circIgfbp2 shRNA lentivirus were effective in HT22 cells for 7 days, respectively. The circIgfbp2 overexpression lentivirus increased circIgfbp2 expression by about 30 folds, while the circIgfbp2 shRNA(sh-circIgfbp2_1) lentivirus decreased circIgfbp2 by around half (*P* < 0.05, Supplementary Fig. 1A, B).

We then treated total RNA extracted from HT22 cells and mice brains with RNase R, and the qRT-PCR results confirmed that the circIgfbp2 overexpression and the circIgfbp2 shRNA lentivirus, targeted specifically the circIgfbp2, not the liner Igfbp2 mRNA (*P* < 0.0001, Supplementary Fig. 1C–H).

**Fig. 4:**
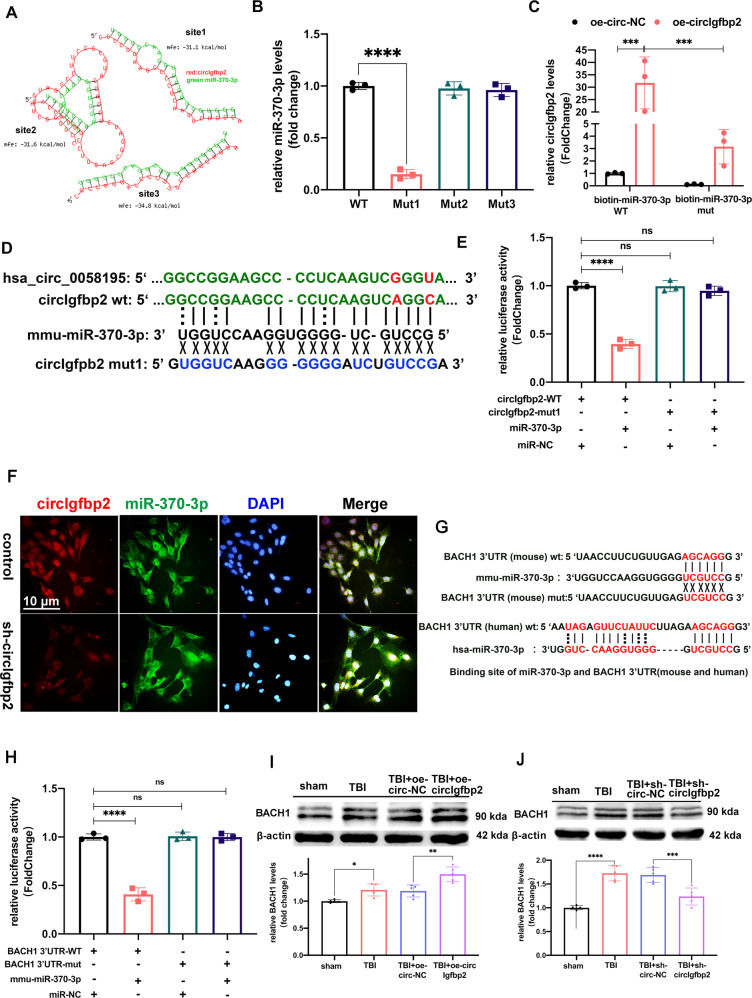
CircIgfbp2 acts as a molecular sponge of miR-370-3p to regulate BACH1. **A** RNAhybrid predicted circIgfbp2 and mmu-miR-370-3p binding sites: site1: mfe (minimum free energy) = −31 kcal/mol, site2: mfe = −31.6 kcal/mol, site3 = −34.8 kcal/mol. **B** The expression of miR-370-3p after biotin-labeled wild-type or mutated circIgfbp2 probe pull-down miR-370-3p in HT22 cells. *n* = 3 replication, ****p < 0.0001, one-way ANOVA followed by Tukey’s multiple comparisons test. **C** The expression of circIgfbp2 after the biotin-labeled miR-370-3p pull-down circIgfbp2, *n* = 3replications, oe-circIgfbp2 + biotin-miR-370-3p(WT) vs. oe-circ-NC + biotin-miR-370-3p(WT), ***p < 0.001; oe-circIgfbp2 + biotin-miR-370-3p(mut) vs.oe-circIgfbp2 + biotin-miR-370-3p(WT), ***p < 0.001. Two-way ANOVA followed by Tukey’s multiple comparisons test. **D** hsa_circ_0058195 have homologous sequences with circIgfbp2 (the same bases in green and different bases in red). The binding site1 of circIgfbp2 with miR-370-3p and the circIgfbp2 mut1 site are shown. **E** Relative luciferase activity in 293 T cells transfected with a plasmid containing wild-type/mut circIgfbp2 and miR-370-3p mimics/miR-NC. *n* = 3 replications, circIgfbp2-WT + miR-370-3p vs. circIgfbp2-WT + miR-NC, ****p < 0.0001, one-way ANOVA followed by Tukey’s multiple comparisons test. **F** FISH probes indicated that circIgfbp2 and mmu-miR-370-3p were enriched at the cytoplasm in HT22 cells. **G** The predicted binding sites of miR-370-3p and BACH1 3’UTR (mouse and human). **H** Relative luciferase activity in 293 T cells transfected with the wild-type/mut BACH1 3’UTR plasmid or miR-370-3p mimics/miR-NC, *n* = 3 replications. BACH1 3’UTR-WT + mmu-miR-370-3p vs. BACH1 3’UTR-WT + mmu-miR-NC, ****p < 0.0001. One-way ANOVA followed by Tukey’s multiple comparisons test. **I** The BACH1 level was analyzed in the injured brain cortex tissue with overexpression circIgfbp2 3 days after TBI in mice by Western blot, *n* = 5 mice per group. TBI vs. sham, *p < 0.05, TBI + oe-circIgfbp2 vs. TBI + oe-circ-NC, **p < 0.01. One-way ANOVA followed by Tukey’s multiple comparisons test. **J** The BACH1 level was analyzed in the injured brain cortex tissue with knockdown circIgfbp2 3 days after TBI in mice by Western blot, *n* = 5 mice per group. TBI vs. sham, ****p < 0.0001, TBI + sh-circIgfbp2 vs. TBI + sh-circ-NC, ***p < 0.001. One-way ANOVA followed by Tukey’s multiple comparisons test. ns: no significance. All data were represented as mean ± SEM.

It is reported that hsa-miR-370-3p is closely related to affective disorders such as depression [35]. And then, we detected the expression of miR-370-3p after overexpression of circIgfbp2 or knockdown of circIgfbp2. The results showed that circIgfbp2 did not affect the transcription of miR-370-3p (Supplementary Fig. 1I–J). The bioinformatics tool RNAhybrid further predicted three potential binding sites between miR-370-3p and circIgfbp2, with a minimum free energy of <−25 kcal/mol (Fig. 4A). In order to determine the binding site, we first designed a biotin-circIgfbp2 probe that avoided the predicted miRNA binding regions to pull down circIgfbp2 and then mutated each predicted binding site in the circIgfbp2 over-expression structure and tested whether it still could bind miR-370-3p. The level of miR-370-3p decreased after mutation at site 1, while mutations at the other two sites had no effect on the level of miR-370-3p (*P* < 0.0001, Fig. 4B). These results suggest that circIgfbp2 adsorbs miR-370-3p at binding site 1 rather than the other two binding sites in HT22 cells. Next, biotin-labeled miR-370-3p was used to verify the direct binding of miR-370-3p to circIgfbp2. The enrichment of wild-type biotin miR-370-3p and circIgfbp2 was higher than that of mutant miR-370-3p (*P* < 0.001, Fig. 4C). The predicted binding site 1 of circIgfbp2 and miR-370-3p is shown in Fig. 4D. In order to verify the targeting effect of miR-370-3p on circIgfbp2, psiCHECK2-circIgfbp2-WT plasmid, mutant plasmid psiCHECK2-circIgfbp2-mut, and miR-370-3p mimics were transfected into 293 T cells, and luciferase reporter gene assay was performed (*P* < 0.0001, Fig. 4E). The results showed that the luciferase activity of miR-370-3p mimics co-transfected with psiCHECK2-circIgfbp2-wt was lower than that in the control group, while the luciferase activity after co-transfection of miR-370-3p mimics and psiCHECK2-circIgfbp2-Mut1 was not significantly different from the control group, indicating that circIgfbp2 could bind miR-370-3p through site 1. The double-FISH experiment showed that circIgfbp2 and miR-370-3p were mainly localized in the cytoplasm (Fig. 4F).

We transfected miR-370-3p mimics and anti-miR-370-3p into HT22 cells to determine whether miR-370-3p had a crucial role in H_2_O_2_-induced neuronal damage. The relative expression of miR-370-3p is shown in Supplementary Fig. 1K. Then, we determined the downstream target protein displaying increased levels in the brain injury tissue that served as an inducer of TBI. Previous studies have shown that the prevention of cell death by HO-1 through antioxidant and anti-inflammatory effects depends on the regulation of BACH1 [10, 13, 14].

The 3’-UTR of the human and mouse BACH1 mRNA contains a conserved miR-370-3p binding site (Fig. 4G). The luciferase reporter assay showed that the luciferase activity of miR-370-3p mimics co-transfected with psiCHECK2-BACH1 3’UTR-wt was lower than that in the control group. In contrast, the luciferase activity of miR-370-3p mimics co-transfected with psiCHECK2-BACH1 3’UTR-mut did not significantly differ from the control group. miR-370-3p inhibited the luciferase activity of the reporter gene containing a wild-type BACH1 mRNA 3’UTR, but not mutant BACH1 mRNA 3’UTR. These data confirmed that BACH1 was a direct target of miR-370-3p (*P* < 0.0001, Fig. 4H). We then co-transduced HT22 cells with the circIgfbp2 overexpression lentivirus and miR-370-3p mimics or circIgfbp2 shRNA lentivirus. The results showed that miR-370-3p acts as a mediator of circIgfbp2 to control BACH1 expression. Also, the overexpression of circIgfbp2 attenuated the decrease of BACH1 expression induced by the miR-370-3p mimics. Accordingly, the knockdown of circIgfbp2 reduced the expression of BACH1 regulated by miR-370-3p (*P* < 0.01, Supplementary Fig. 1L, M).

Furthermore, to determine whether BACH1 has a crucial role in TBI or not. Western blot was performed to detect the expression of BACH1 in the brain lesions in mice. The results showed that compared with the TBI + circ-NC group, the overexpression of circIgfbp2 promoted the expression of BACH1. The knockdown of circIgfbp2 decreased the expression of BACH1 (*P* < 0.01, Fig. 4I, J). Moreover, the expression of hsa_circ_0058195 was increased in patients with acute TBI (*P* < 0.001, Supplementary Fig. 2A). In addition, we observed that the expression of BACH1 and HO-1proteins was increased, while the expression of PSD95 and Syn proteins was reduced in the brain lesions of the patients suffering acute TBI (*P* < 0.01, Supplementary Fig. 2B, C).

To sum up, these results indicated that BACH1 is probably involved in TBI pathogenesis, and miR-370-3p acted as a mediator of circIgfbp2 to control BACH1 expression.

### MiR-370-3p regulates BACH1 to alleviate mitochondrial dysfunction and oxidative stress-induced synapse dysfunction

**Fig. 5:**
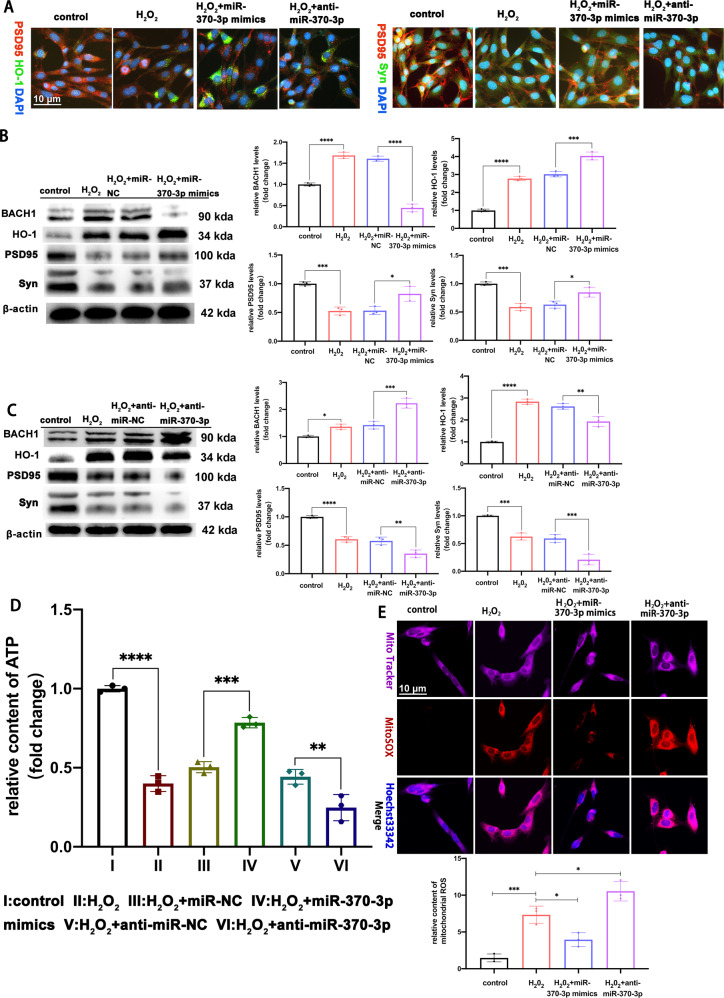
miR-370-3p regulates BACH1 to alleviate mitochondrial dysfunction and oxidative stress-induced synapse dysfunction. **A** The double immunofluorescence staining showed the colocalization of PSD95 and HO-1, PSD95 and Syn in control, H_2_O_2_, H_2_O_2_ + miR-370-3p mimics, and H_2_O_2_ + anti-miR-370-3p group. **B** The expression of BACH1, HO-1, PSD95 and Syn proteins were analyzed in the HT22 cells transduced with miR-NC or miR-370-3p mimics for 48 h and treated with H_2_O_2_ (600 µmol/L) for 6 h by Western blot, *n* = 3 replication. BACH1: H_2_O_2_ vs. control, ****p < 0.0001, H_2_O_2_ + miR-370-3p mimics vs. H_2_O_2_ + miR-NC, ****p < 0.0001,;HO-1: H_2_O_2_ vs. control, ****p < 0.0001, H_2_O_2_ + miR-370-3p mimics vs. H_2_O_2_ + miR-NC, ***p < 0.001,;PSD95: H_2_O_2_ vs. control, ***p < 0.001, H_2_O_2_ + miR-370-3p mimics vs. H_2_O_2_ + miR-NC, *p < 0.05; Syn: H_2_O_2_ vs. control, ***p < 0.001, H_2_O_2_ + miR-370-3p mimics vs. H_2_O_2_ + miR-NC, *p < 0.05. One-way ANOVA followed by Tukey’s multiple comparisons test. **C** The expression of BACH1, HO-1, PSD95 and Syn were analyzed in the HT22 cells transduced with anti-miR-NC or anti-miR-370-3p for 48 h and treated with H_2_O_2_ (600 µmol/L) for 6 h by Western blot, *n* = 3 replications.BACH1: H_2_O_2_ vs. control, *p < 0.05, H_2_O_2_ + anti-miR-370-3p vs. H_2_O_2_ + anti-miR-NC, ***p < 0.001;HO-1; H_2_O_2_ vs. control, ****p < 0.0001,H_2_O_2_ + anti-miR-370-3p vs. H_2_O_2_ + anti-miR-NC, **p < 0.01; PSD95: H_2_O_2_ vs. control, ****p < 0.0001, H_2_O_2_ + anti-miR-370-3p vs. H_2_O_2_ + anti-miR-NC, **p < 0.01; Syn: H_2_O_2_ vs. control, ***p < 0.001, H_2_O_2_ + anti-miR-370-3p vs. H_2_O_2_ + anti-miR-NC, ***p < 0.001. One-way ANOVA followed by Tukey’s multiple comparisons test. **D** The content of ATP with miR-370-3p overexpression or knockdown in HT22 cells treated with H_2_O_2_ (600 µmol/L) for 6 h, *n* = 3 replications.H_2_O_2_ vs. control, ****p < 0.0001, H_2_O_2_ + miR-370-3p mimics vs. H_2_O_2_ + miR-NC, ***p < 0.001, H_2_O_2_ + anti-miR-370-3p vs. H_2_O_2_ + anti-miR-NC, **p < 0.01. One-way ANOVA followed by Tukey’s multiple comparisons test. **E** Representative fluorescent staining of Mitosox for mitochondrial ROS with miR-370-3p overexpression or knockdown in HT22 cells treated with H_2_O_2_ (600 µmol/L) for 6 h, and quantitative analysis of the mean optical density analysis of Mitosox, *n* = 3 replications. H_2_O_2_ vs. control,***p < 0.001,H_2_O_2_ + miR-370-3p mimics vs. H_2_O_2_, *p < 0.05,H_2_O_2_ + anti-miR-370-3p vs. H_2_O_2_, *p < 0.05, one-way ANOVA followed by Tukey’s multiple comparisons test. All data were represented as mean ± SEM.

In order to investigate whether miR-370-3p could alleviate mitochondrial dysfunction and oxidative stress-induced synapse dysfunction by targeting BACH1, we transfected miR-370-3p mimics or miR-370-3p inhibitors into HT22 cells for 48 h and then treated the cells with H_2_O_2_ (600 μmol/L) for 6 h. We detected the co-localization and expression levels of BACH1, HO-1, PSD95, and Syn by double immunofluorescent staining (Fig. 5A) and Western blot. MiR-370-3p mimics transfection reduced H_2_O_2_-induced mitochondrial oxidative stress-mediated synapse dysfunction, inhibited the expression of BACH1, and increased the expression of HO-1, PSD95, and Syn (*P* < 0.05, Fig. 5B). Transfection with anti-miR-370-3p increased H_2_O_2_-induced mitochondrial oxidative stress-mediated synapse dysfunction, aggravated the expression of BACH1, and reduced the expression of HO-1, PSD95, and Syn (*P* < 0.05, Fig. 5C). Meanwhile, in H_2_O_2_-treated HT22 cells, transfection with miR-370-3p mimics increased the mitochondrial ATP content and decreased the mitochondrial ROS content, while the results of HT22 cells with anti-miR-370-3p treatment showed aggravated mitochondrial oxidative stress (*P* < 0.05, Fig. 5D, E). Therefore, the results suggested that miR-370-3p regulated BACH1 to alleviate mitochondrial dysfunction and oxidative stress-induced synapse dysfunction.

### Knocking down circIgfbp2 enhances mitochondrial antioxidant stress capacity through the circIgfbp2/miR-370-3p/BACH1/HO-1 signaling axis

**Fig. 6:**
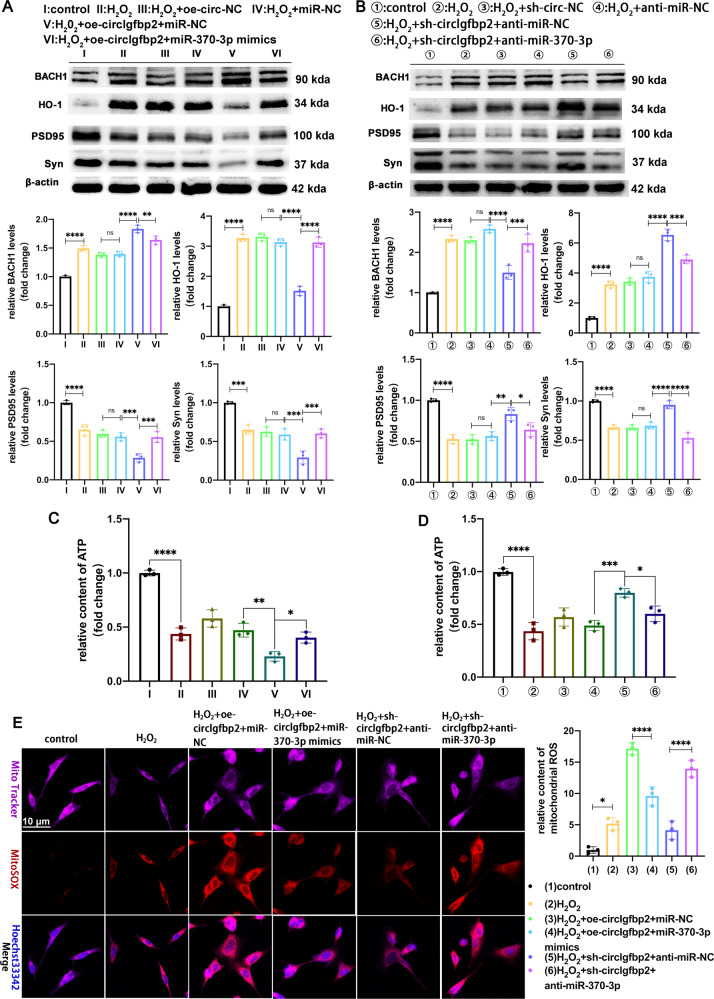
Silencing of circIgfbp2 regulates circIgfbp2/miR-370-3p/BACH1 axis to alleviate mitochondrial dysfunction and oxidative stress-induced synapse dysfunction. **A** Western blot analysis of BACH1, HO-1, PSD95 and Syn in HT22 cells transduced with oe-circIgfbp2 lentivirus for 7 days, and then transduced with miR-NC or miR-370-3p mimics for 48 h, finally H_2_O_2_ (600 µmol/L) treated HT22 cells for 6 h. The results showed that transfection with oe-circIgfbp2 alone aggravated H_2_O_2_-induced mitochondrial oxidative stress-mediated synapse dysfunction, the expression of BACH1was upregulated, and HO-1, PSD95 and Syn were downregulated. The co-transfection with miR-370-3p mimics reversed these effects. *n* = 3 replications. BACH1:H_2_O_2_ + oe-circIgfbp2+miR-NC vs. H_2_O_2_ + miR-NC, ****p < 0.0001; H_2_O_2_ + oe-circIgfbp2+miR-370-3p mimics vs. H_2_O_2_ + oe-circIgfbp2+miR-NC, **p < 0.01; HO-1:H_2_O_2_ + oe-circIgfbp2+miR-NC vs. H_2_O_2_ + miR-NC, ****p < 0.0001;H_2_O_2_ + oe-circIgfbp2+miR-370-3p mimics vs. H_2_O_2_ + oe-circIgfbp2+miR-NC, ****p < 0.0001; PSD95: H_2_O_2_ + oe-circIgfbp2+miR-NC vs. H_2_O_2_ + miR-NC, ***p < 0.001, H_2_O_2_ + oe-circIgfbp2+miR-370-3p mimics vs. H_2_O_2_ + oe-circIgfbp_2_ + miR-NC, ***p < 0.001; Syn: H_2_O_2_ + oe-circIgfbp2+miR-NC vs. H_2_O_2_ + miR-NC, ***p < 0.001, H_2_O_2_ + oe-circIgfbp2+miR-370-3p mimics vs. H_2_O_2_ + oe-circIgfbp2+miR-NC, ***p < 0.001; one-way ANOVA followed by Tukey’s multiple comparisons test. **B** Western blot analysis of BACH1, HO-1, PSD95, Syn in HT22 cells transduced with circIgfbp2 shRNA lentivirus cells for 7 days, and then transduced with anti-miR-NC or anti-miR-370-3p for 48 h, finally treated with H_2_O_2_ (600 µmol/L) for 6 h. The transfection with sh-circIgfbp2 alone attenuated H_2_O_2_-induced mitochondrial oxidative stress-mediated synapse dysfunction, the expression of BACH1was downregulated; HO-1, PSD95 and Syn were upregulated, while co-transfection with anti-miR-370-3p reversed these effects, *n* = 3 replications. BACH1: H_2_O_2_ + sh-circIgfbp2+anti-miR-NC vs. H_2_O_2_ + anti-miR-NC, ****p  <  0.0001, H_2_O_2_ + sh-circIgfbp2+anti-miR-370-3p vs. H_2_O_2_ + sh-circIgfbp_2_ + anti-miR-NC, ***p < 0.001; HO-1: H_2_O_2_ + sh-circIgfbp2+anti-miR-NC vs. H_2_O_2_ + anti-miR-NC, ****p < 0.0001, H_2_O_2_ + sh-circIgfbp2+anti-miR-370-3p vs. H_2_O_2_ + sh-circIgfbp2+anti-miR-NC, ***p < 0.001; PSD95: H_2_O_2_ + sh-circIgfbp2+anti-miR-NC vs. H_2_O_2_ + anti-miR-NC, **p < 0.01, H_2_O_2_ + sh-circIgfbp2+anti-miR-370-3p vs. H_2_O_2_ + sh-circIgfbp2+anti-miR-NC, *p < 0.05; Syn: H_2_O_2_ + sh-circIgfbp2+anti-miR-NC vs. H_2_O_2_ + anti-miR-NC, ****p < 0.0001, H_2_O_2_ + sh-circIgfbp2+anti-miR-370-3p vs. H_2_O_2_ + sh-circIgfbp2+anti-miR-NC,****p < 0.0001 one-way ANOVA followed by Tukey’s multiple comparisons test. **C** Transduction of HT_22_ cells with the miR-370-3p mimics significantly reversed the oe-circIgfbp2 -induced decrease the content of ATP, *n* = 3 replications. H_2_O_2_ + oe-circIgfbp2+miR-NC vs. H_2_O_2_ + miR-NC, **p < 0.01, H_2_O_2_ + oe-circIgfbp2+miR-370-3p mimics vs. H_2_O_2_ + oe-circIgfbp2+miR-NC, *p < 0.05; one-way ANOVA followed by Tukey’s multiple comparisons test. **D** Transduction of HT22 cells with the anti-miR-370-3p mimics significantly attenuated the sh-circIgfbp2 -induced increase the content of ATP. *n* = 3 replications. H_2_O_2_ + sh-circIgfbp2+anti-miR-NC vs. H_2_O_2_ + anti-miR-NC, ***p < 0.001, H_2_O_2_ + sh-circIgfbp2+anti-miR-370-3p vs. H_2_O_2_ + sh-circIgfbp2+anti-miR-NC, *p < 0.05.One-way ANOVA followed by Tukey’s multiple comparisons test. **E** Transduction of HT22 cells with the miR-370-3p mimics significantly reversed the oe-circIgfbp2-induced increase the content of mitochondrial ROS; transduction of HT22 cells with the anti-miR-370-3p significantly attenuated the sh-circIgfbp2-induced the decrease of mitochondrial ROS, *n* = 3 replications. H_2_O_2_ + oe-circIgfbp2+miR-370-3p mimics vs. H_2_O_2_ + oe-circIgfbp2+miR-NC, ****p < 0.0001, H_2_O_2_ + sh-circIgfbp2+anti-miR-370-3p vs. H_2_O_2_ + sh-circIgfbp2+anti-miR-NC, ****p < 0.0001;one-way ANOVA followed by Tukey’s multiple comparisons test. ns: no significance. All data were represented as mean ± SEM.

In order to analyze whether circIgfbp2 could affect the recovery of mitochondrial function by regulating the miR-370-3p/BACH1/HO-1 signaling axis, we designed a rescue experiment. Briefly, H_2_O_2_-treated HT22 cells were transfected with oe-circIgfbp2 or miR-370-3p mimics or sh-circIgfbp2 or anti-miR-370-3p, either alone or in combination. The results showed that transfection with oe-circIgfbp2 alone aggravated H_2_O_2_-induced mitochondrial oxidative stress-mediated synapse dysfunction; the expression of BACH1 was up-regulated while HO-1, PSD95, and Syn were downregulated. The co-transfection with miR-370-3p mimics reduced the negative effects of circIgfbp2 overexpression (Fig. 6A). The transfection with sh-circIgfbp2 alone decreased H_2_O_2_-induced mitochondrial oxidative stress-mediated synapse dysfunction; the expression of BACH1 was downregulated, and HO-1, PSD95, Syn were up-regulated. Co-transfection with anti-miR-370-3p rescued these effects of sh-circIgfbp2 (Fig. 6B).

We next detected the content of ATP and ROS in mitochondrial in H_2_O_2_-treated HT22 cells. The circIgfbp2 overexpression alone increased the content of mitochondrial ROS and reduced the content of ATP. The co-transfection with miR-370-3p mimics attenuated the negative effects of circIgfbp2 overexpression. (Fig. 6C). The transfection with sh-circIgfbp2 alone attenuated H_2_O_2_-induced mitochondrial dysfunction; the ROS content was downregulated, and ATP content was up-regulated. Co-transfection with anti-miR-370-3p reversed these effects of sh-circIgfbp2 (Fig. 6D, E).

The results revealed that mitochondrial antioxidant stress capacity was enhanced after knocking down circIgfbp2, and this effect was mediated through the circIgfbp2/miR-370-3p/BACH1/HO-1 signaling axis.

## Discussion

In this study, we discovered a novel circular RNA, circIgfbp2, linking neural plasticity and anxiety after traumatic brain injury. Firstly, we obtained differentially expressed circRNAs in TBI mice by RNA-seq, which was followed by bioinformatics analysis screening and generated the target circRNA, mmu_circ_0008937 (host gene *Igfbp2*). Next, we compared the sequences of mmu_circ_0008937 with the human circRNAs database by BLAST, resulting in hsa_circ_0058195 (host gene also *IGFBP2*) with 92.7% identity with mmu_circ_0008937. Subsequently, our data suggested that the high levels of hsa_circ_0058195 in the serum of acute TBI patients might be associated with anxiety. Therefore, these two circRNAs (mmu_circ_0008937 and hsa_circ_0058195) derived from homologous genes (I*gfbp2*/*IGFBP2*) might be associated with anxiety after TBI (Supplementary Fig. 3).

Our subsequent behavioral experiments in mice further showed that mmu_circ_0008937 was associated with anxiety-like behavior and sleep disorders after TBI. The latest studies show that mitochondrial oxidative stress can lead to anxiety [36]. To investigate the potential mechanisms of mmu_circ_0008937 affecting anxiety behavior, we cultured hippocampal neurons (HT22 cell line) and performed intervention by H_2_O_2_ to mimic neurological impairment by oxidative stress after TBI as previously reported [37, 38]. Our data suggested that inhibition of circIgfbp2 alleviated mitochondrial dysfunction and oxidative stress-induced synapse dysfunction after TBI through the miR-370-3p/BACH1/HO-1 axis.

Deep sleep induced by chloral hydrate is dominated by delta waves, which is related to the fourth phase of non-rapid eye movement (NREM) sleep [39]. Several clinical studies have already confirmed patients’ impaired delta waves in sleep EEG after TBI [40, 41], which is consistent with our results in TBI mice. Our results further showed that the delta waves were impaired not only in the ipsilesional cortex but also in the contralesional cortex on the 30th day after TBI. These results suggested that the damage of EEG activity after TBI was not limited to the ipsilesional hemisphere, but the whole brain may be affected. In addition, circIgfbp2 mainly affected the delta wave of ipsilateral EEG but had no significant effect on the contralateral delta wave.

CircRNAs are small RNAs abundant in the brain and expressed in complex spatiotemporal patterns that have essential roles in the central nervous system. It has been shown that circRNAs are highly enriched in the brains of a variety of mammals, including mice, primates, and humans [42, 43]. CircRNAs dominate the number of the 1000 most abundant RNA transcripts in the primate brain [44]. The abnormal expression of circStag1 and circHipk2 has been associated with astrocyte dysfunction and depressive symptoms [45]. Zhang et al. found that the overexpression of circStag1 and knockout of circHipk2 could inhibit the progression of depression [46]. Moreover, circTfrc and circTnik are up-regulated in depression patients, which suggests that the functions of circTfrc and circTnik might be related to the pathogenesis of depression [47]. Furthermore, preclinical studies discovered that the brain circRNA profiles were significantly altered after TBI [21, 22] and that some circRNAs were involved in mood disorders [24–26]. Our results showed that mmu_ circ_0008937 was associated with anxiety-like behavior and sleep disorders after TBI.

CircRNA, as an important component of ncRNA, can regulate neural functions and act as a miRNA sponge to indirectly regulate miRNA target genes’ expression by competitively binding to miRNAs in nervous diseases [48]. The circRNA of human CDR1as/ciRS-7 functions as a miR-7 sponge; it harbors 74 binding sites for miR-7 and can bind to the argonaute (AGO) protein in a miR-7-dependent manner. Binding to the AGO protein allows the circRNA of human CDR1as/ciRS-7 to participate in the initiation and progression of Parkinson’s disease [49]. Interestingly, other circRNAs are also involved in depression-like behaviors. Upregulation of circDYM expression reduces depression-like behaviors, which may be mediated by circDYM inhibiting miR-9 activity as an endogenous miR-9 sponge, resulting in reduced microglia activation through deactivation of heat shock protein 90 (Hsp90) ubiquitination [50]. In the present study, we used bioinformatics analysis to predict whether circIgfbp2 sponges miR-370-3p regulate mitochondrial oxidative stress-induced synapse dysfunction-related genes [51–53]. CircIgfbp2 and miR-370-3p were mainly found in the cytoplasm of HT22 cells; thus, circIgfbp2 might exert a biological function by adsorbing miR-370-3p. Using a double luciferase reporter gene assay, we confirmed that circIgfbp2 could bind to miR-370-3p. Besides, miR-370-3p was significantly reduced after overexpression of circIgfbp2 and significantly elevated after its knockdown in H_2_O_2_-treated HT22 cells. These results confirm that circIgfbp2, as a ceRNA, regulates miR-370-3p.

CircRNAs are involved in mitochondrial function. Mitochondrial oxidative stress and mitochondrial dysfunction have been shown to participate in mood disorders [54–56]. It is widely accepted that synaptic damage is likely caused by mitochondrial oxidative stress and mitochondrial dysfunction [5, 9]. During the process of electron transfer, including oxidative phosphorylation produced during ATP synthesis [57], mitochondria produce large amounts of ROS that attack the cell membrane and the organelles, causing membrane lipid peroxidation, mitochondrial membrane potential decreasing [58, 59], membrane permeability-increasing [60, 61]. A previous study found that knockout of circRNA_0084043 could significantly reduce oxidative stress; circRNA_0084043 functions by sponging miR-140-3p, thus reducing the content of MDA in mitochondria and enhancing the activities of SOD and GPXα [62]. In this study, the knockdown of circIgfbp2 alleviated mitochondrial oxidative stress and protected synapses to relieve anxiety and sleep disorders after TBI. In addition, synaptic damage can further contribute to affective disorders [63, 64]. So, improving the ability of cells to resist mitochondrial oxidative stress might significantly reduce neuron damage and protect synapses from affective disorders after TBI. In this study, we found that knockdown of circIgfbp2 increased HO-1 and alleviated the loss of PSD95 and Syn after TBI, while overexpression of circIgfbp2 aggravated these changes. These data indicate that circIgfbp2 knockdown reduces anxiety and promotes neurological recovery by decreasing mitochondrial oxidative stress after TBI.

BACH1 has an essential role in the mitochondrial oxidative stress and inflammatory response of TBI [65, 66]. The expression of HO-1, an Nrf2-regulated cytoprotective enzyme, has been proven to be altered after TBI and cerebral ischemia [65–67]. BACH1 is a transcriptional repressor of the HO-1 gene that has a critical role in protecting tissue from oxidative stress [12, 68]. A previous study reported that BACH1 could inhibit mitochondrial metabolism through transcriptional suppression of mitochondrial membrane genes and enhance mitochondrial respiratory inhibition [11]. Furthermore, HO-1 and its catalytic products can protect tissues and cells through anti-oxidation and anti-inflammation [69]. In this study, based on the prediction algorithms and argonaute2 (AGO2) CLIP-Seq results in the Starbase database [70], we predicted that miR-370-3p could bind to BACH1 mRNA 3’UTR to regulate the expression of BACH1. Our experiments in HT22 cells further confirmed that transfection of miR-370-3p mimics could downregulate the expression of BACH1, while transfection of anti-miR-370-3p could up-regulate the expression of BACH1. Meanwhile, the double-luciferase reporter gene assay confirmed the complementary binding of miR-370-3p and BACH1 mRNA 3’UTR. To sum up, these results indicated that BACH1 was probably involved in TBI pathogenesis, and miR-370-3p acted as a mediator of circIgfbp2 to control BACH1 expression.

In conclusion, this is the first study that reported on circIgfbp2 knockdown relieving anxiety-like behaviors induced by synaptic damage after TBI. Inhibition of circIgfbp2 alleviated mitochondria oxidative stress depending on sponge miR-370-3p and subsequently targeted BACH1 to enhance the expression of HO-1. Our results lay a foundation for future studies on the function and mechanism of circRNA in the occurrence and development of TBI and hopefully provide a new potential molecular target for mood disorders after TBI.

## Supplementary information

Supplementary Table 1

Supplementary Table 2

Supplementary Table 3

Supplementary Table 4

Supplementary figures and the legends

Supplementary Fig. 1

Supplementary Fig. 2

Supplementary Fig. 3
