# Supplementary material for: A novel circular RNA, circIgfbp2, links neural plasticity and anxiety through targeting mitochondrial dysfunction and oxidative stress-induced synapse dysfunction after traumatic brain injury
Source: Mol Psychiatry. 2022 Aug 2;27(11):4575–89. doi: 10.1038/s41380-022-01711-7 (PMC9734054; doi:10.1038/s41380-022-01711-7)
Supplement: Supplementary file 1 — Supplementary Table 1 [file 41380_2022_1711_MOESM1_ESM.docx]

**Supplementary Table 1.** Clinical data from control and TBI cases.

| **NO.** | **Age** | **gender** | **time after TBI** | **Contusion site** | **GCS** | **GOS** |
| --- | --- | --- | --- | --- | --- | --- |
| **1** | **38** | **F** | **NA** | **NA** | **15** | **5** |
| **2** | **45** | **M** | **NA** | **NA** | **15** | **5** |
| **3** | **52** | **M** | **NA** | **NA** | **15** | **5** |
| **4** | **47** | **F** | **10h** | **Frontotemporal(Bilateral)** | **7** | **3** |
| **5** | **36** | **M** | **21h** | **Frontotemporal(LT)** | **8** | **4** |
| **6** | **42** | **M** | **8h** | **Frontotemporal(LT)** | **7** | **3** |
| **7** | **32** | **F** | **17h** | **Frontal(Bilateral)** | **9** | **4** |
| **8** | **43** | **F** | **14h** | **Frontal(RT)** | **8** | **4** |
| **9** | **52** | **M** | **19h** | **Temporal(RT)** | **7** | **4** |
