# Supplementary material for: A novel circular RNA, circIgfbp2, links neural plasticity and anxiety through targeting mitochondrial dysfunction and oxidative stress-induced synapse dysfunction after traumatic brain injury
Source: Mol Psychiatry. 2022 Aug 2;27(11):4575–89. doi: 10.1038/s41380-022-01711-7 (PMC9734054; doi:10.1038/s41380-022-01711-7)
Supplement: Supplementary file 3 — Supplementary Table 3 [file 41380_2022_1711_MOESM3_ESM.docx]

**Supplementary Table 3.** The sequences of sh-circIgfbp2.

| **Target** | **Sequence (5’ → 3’)** |
| --- | --- |
| **sh-circIgfbp2_ 1** | GTACAACCTTAAGCAGACAGT |
| **sh-circIgfbp2_2** | GCCTTAAGCAGACAGTGATGA |
| **sh-circIgfbp2_3** | GCAGACAGTGATGACGACCAC |
| **sh-circ-NC** | CAGTAGTGACAGACGAATTCC |
