# Supplementary material for: A novel circular RNA, circIgfbp2, links neural plasticity and anxiety through targeting mitochondrial dysfunction and oxidative stress-induced synapse dysfunction after traumatic brain injury
Source: Mol Psychiatry. 2022 Aug 2;27(11):4575–89. doi: 10.1038/s41380-022-01711-7 (PMC9734054; doi:10.1038/s41380-022-01711-7)
Supplement: Supplementary file 4 — Supplementary Table 4 [file 41380_2022_1711_MOESM4_ESM.docx]

**Supplementary Table 4.** The primers for qRT-PCR.

| **Target** | **Sequence (5’ → 3’)** |
| --- | --- |
| **circIgfbp2 (divergent)** | F: ACAACCTTAAGCAGACAGTGATG  R: TTGACCTTCTCCCGGAACAC |
| **circIgfbp2 (convergent)** | F: ATAGGTCATCTAGGGGGT  R: CGAGATGGTCATCCGCTC |
| **Igfbp2** | F: CTTAAGCAGTGCAAGATG TCT C  R: CTGCTCGTTGTAGAAGAGATGG |
| **BACH1** | F: TTCATGCTTCTGTT  R: CAGCCAA |
| **hsa_circ_0058195** | F: ACAACCTTAAGCAGACAGTGATG  R: TTGACCTTCTCCCGGAACAC |
| **GAPDH (mouse)** | F: AGGTCGGTGTGAACGGATTTG  R: TGTAGACCATGTAGTTGAGGTCA |
| **GAPDH (human)** | F: GGAGCGAGATCCCTCCAAAAT  R: GGCTGTTGTCATACTTCTCATGG |
