# Supplementary material for: A novel circular RNA, circIgfbp2, links neural plasticity and anxiety through targeting mitochondrial dysfunction and oxidative stress-induced synapse dysfunction after traumatic brain injury
Source: Mol Psychiatry. 2022 Aug 2;27(11):4575–89. doi: 10.1038/s41380-022-01711-7 (PMC9734054; doi:10.1038/s41380-022-01711-7)
Supplement: Supplementary file 5 — Supplementary figures and the legends [file 41380_2022_1711_MOESM5_ESM.docx]

**Supplementary figures and the legends**

**
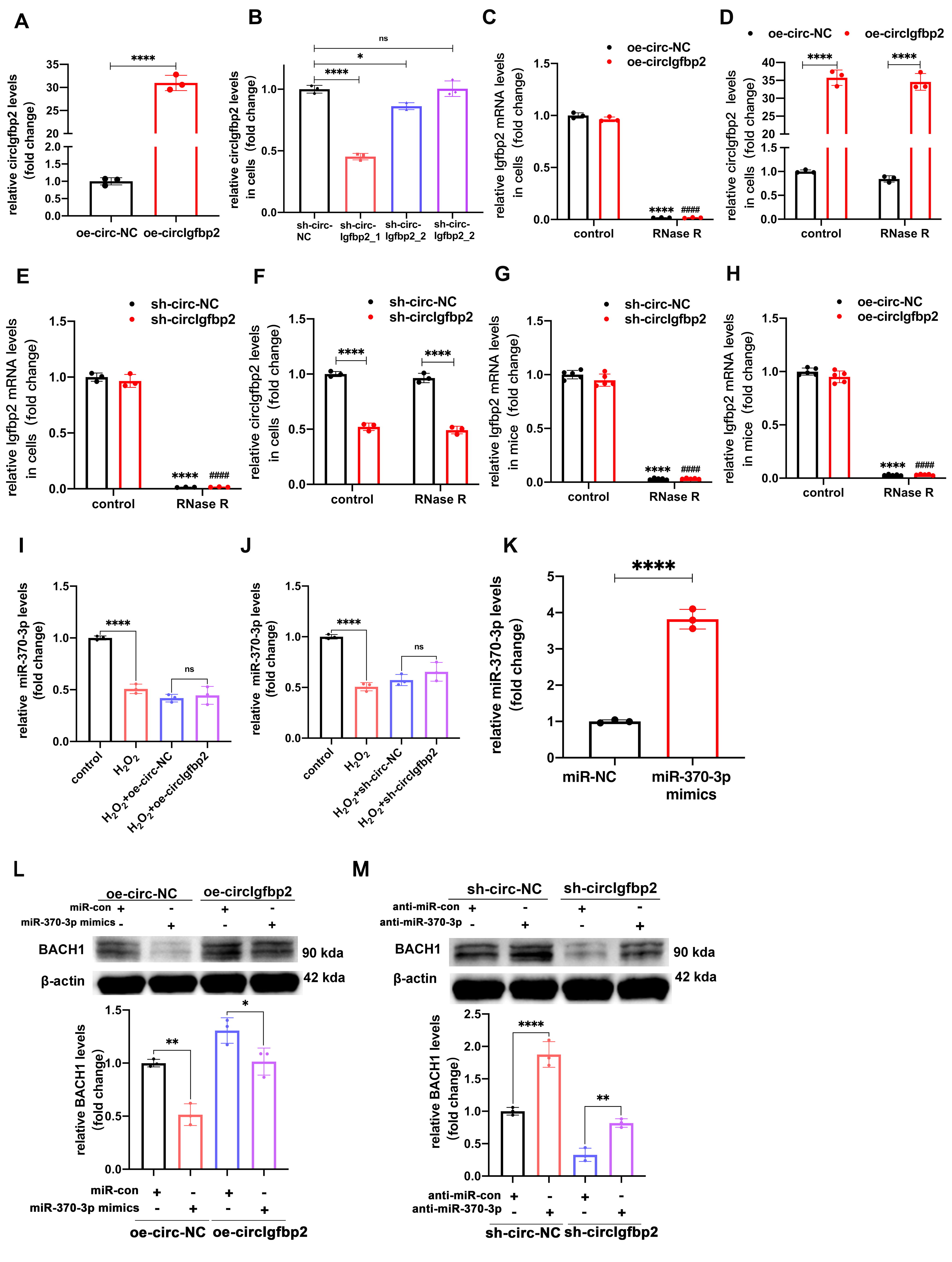
** **Supplementary Fig. 1 CircIgfbp2 regulates BACH1 by binding to miR-370-3p. (A)** Relative expression of circIgfbp2 in HT22 cells after oe-circIgfbp2 lentivirus transduction for 7 days, as determined by qRT-PCR. n=3 replications, ****p<0.0001, two-tailed t-test. **(B)** Relative expression of circIgfbp2 in HT22 cells after circIgfbp2 shRNA lentivirus transduction for 7 days, as determined by qRT-PCR. n=3 replications, sh-circIgfbp2_1 vs. sh-circ-NC, ****p<0.0001;sh-circIgfbp2_2 vs. sh-circ-NC, *p<0.05; sh-circIgfbp2_3 vs. sh-circ-NC, p>0.05, ns, one-way ANOVA followed by Tukey’s multiple comparisons test. **(C)** Relative expression of the Igfbp2 mRNA in HT22 cells after oe-circIgfbp2 lentivirus transduction by RnaseR, as measured by qRT-PCR. n=3 replications. Rnase R+oe-circ-NC vs. control+oe-circ-NC, ****p<0.0001, Rnase R+oe-circIgfbp2 vs. control+oe-circIgfbp2, ^####^p<0.0001. Two-way ANOVA followed by Tukey’s multiple comparisons test. **(D)** Relative expression of the circIgfbp2 in HT22 cells after oe-circIgfbp2 lentivirus transduction by RnaseR, as measured by qRT-PCR. n=3 replications. Rnase R+oe-circ-NC vs. control+oe-circ-NC, ****p<0.0001, Rnase R+oe-circIgfbp2 vs. control+oe-circIgfbp2, ****p<0.0001, two-way ANOVA followed by Tukey’s multiple comparisons test. **(E)**Relative expression of the Igfbp2 mRNA in HT22 cells after circIgfbp2 shRNA lentivirus transduction by Rnase R, as measured by qRT-PCR. n=3 replications. Rnase R+sh-circ-NC vs. control+sh-circ-NC, ****p<0.0001, Rnase R+sh-circIgfbp2 vs. control+sh-circIgfbp2, ^####^p<0.0001, two-way ANOVA followed by Tukey’s multiple comparisons test. **(F)** Relative expression of the circIgfbp2 in HT22 cells after circIgfbp2 shRNA lentivirus transduction by Rnase R, as measured by qRT-PCR. n=3 replications. Rnase R+sh-circ-NC vs. control+sh-circ-NC, ****p<0.0001, Rnase R+oe-circIgfbp2vs.control+oe-circIgfbp2, ****p<0.0001, two-way ANOVA followed by Tukey’s multiple comparisons test. **(G)** The relative expression of the Igfbp2 mRNA in mice after oe-circIgfbp2 lentivirus infection followed RnaseR digestion, as measured by qRT-PCR. n=5 mice per group. Rnase R+oe-circ-NC vs. control+oe-circ-NC, ****p<0.0001, Rnase R+oe-circIgfbp2 vs. control+oe-circIgfbp2, ^####^p<0.0001. Two-way ANOVA followed by Tukey’s multiple comparisons test. **(H)** The relative expression of the Igfbp2 mRNA in mice after circIgfbp2 shRNA lentivirus infection followed Rnase R digestion, as measured by qRT-PCR. n=5 mice per group. Rnase R+sh-circ-NC vs. control+sh-circ-NC, ****p<0.0001, Rnase R+sh-circIgfbp2 vs. control+sh-circIgfbp2, ^####^p<0.0001, two-way ANOVA followed by Tukey’s multiple comparisons test. **(I-J)** The expression levels of miR-370-3p in H_2_O_2_ induced HT22 cells were detected by qRT-PCR with oe-or sh-circIgfbp2 for 7 days. n=3 replication. For oe-circIgfbp2, H_2_O_2_ vs.control, ****p<0.0001; H_2_O_2_ +oe-circIgfbp2 vs. H_2_O_2_+oe-circ-NC, p>0.05, ns. For sh-circIgfbp2, H_2_O_2_ vs. control,****p<0.0001; H_2_O_2_+sh-circIgfbp2 vs. H_2_O_2_ +sh-circ-NC, p>0.05, ns. Two-way ANOVA followed by Tukey’s multiple comparisons test. **(K)** The expression of miR-370-3p after HT22 cells were transduced with miR-NC or miR-370-3p mimics for 48h, measured by qRT-PCR, n=3 replications. miR-370-3p mimics vs. miR-NC, ****p<0.0001. Two-tailed t-test. **(L)** The expression of BACH1 was analyzed in the HT22 cells transduced with oe-circIgfbp2 lentivirus for 7 days, and transduced with miR-370-3p mimics or miR-NC for 48 h by Western blot, n=3 replication. oe-circ-NC+miR-370-3p mimics vs. oe-circ-NC+miR-con, **p<0.01; oe-circIgfbp2+miR-370-3p mimics vs. oe-circIgfbp2+miR-con, *p<0.05. One-way ANOVA followed by Tukey’s multiple comparisons test. **(M)** The expression of BACH1 was analyzed in the HT22 cells transduced with circIgfbp2 shRNA lentivirus for 7 days, and transduced with anti-miR-370-3p or anti-miR-NC for 48 h followed Western blot, n=3 replication. Sh-circ-NC+anti-miR-370-3p vs. sh-circ-NC+anti-miR-con, ****p<0.0001; sh-circIgfbp2+anti-miR-370-3p vs. sh-circIgfbp2+miR-con, **p<0.01. One-way ANOVA followed by Tukey’s multiple comparisons test. ns: no significance. All data were represented as mean ± SEM.


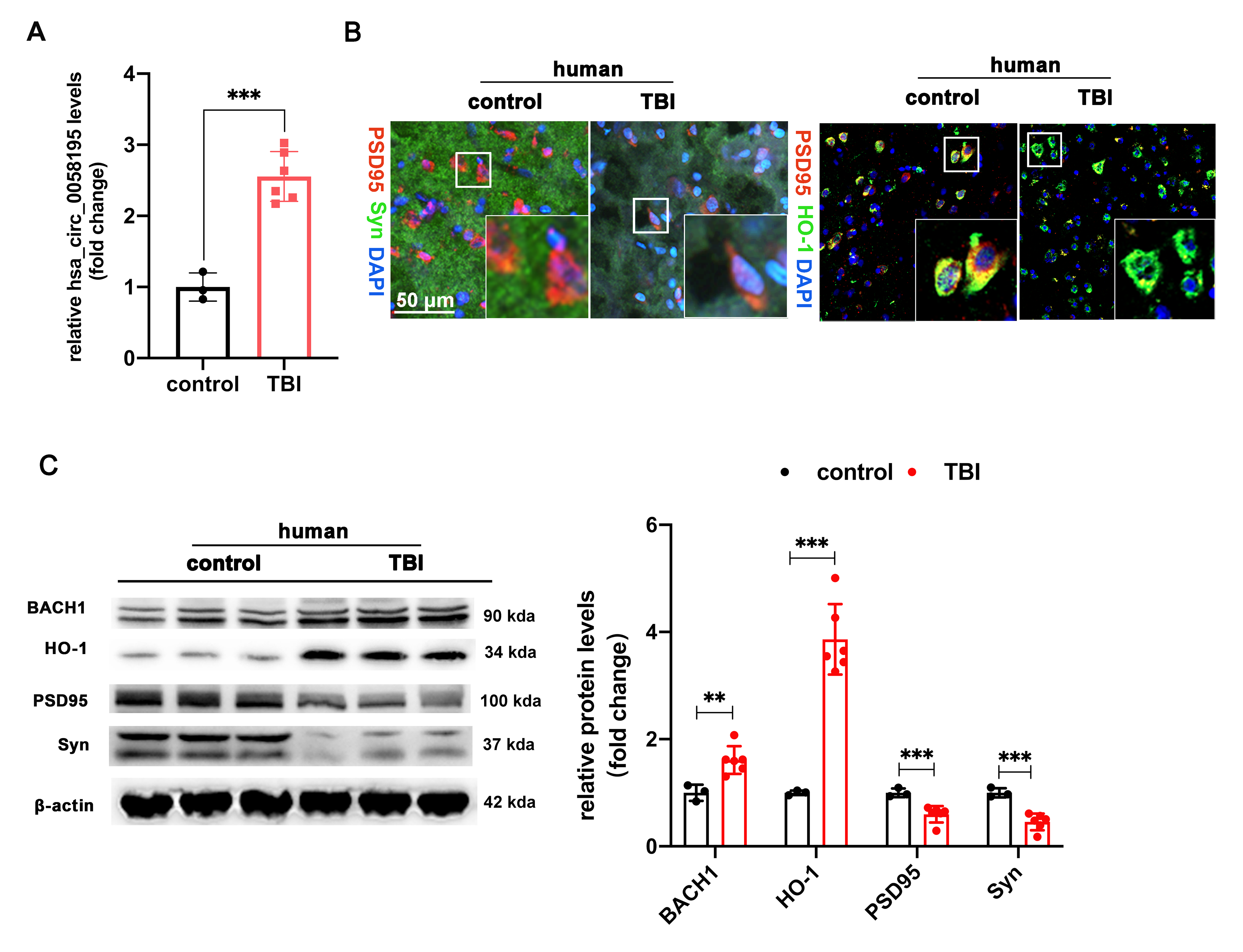
 **Supplementary Fig. 2 The expression of has_circ_0058195 and relative proteins in TBI patients. (A)** The expression levels of has_circ_0058195 in the brain specimens of patients with acute TBI, TBI patients n= 6, control patients n=3. TBI vs. control, ***p<0.001. **(B)** The co-localization of PSD95 with Syn and HO-1 was detected by double immunofluorescence in patients with acute TBI. One-way ANOVA followed by Tukey’s multiple comparisons test. **(C)** The expression of BACH1, HO-1, PSD95, and Syn proteins were analyzed in patients with acute TBI by Western blot. TBI patients (n=6), control patients (n=3), BACH1: TBI vs. sham, **p<0.01; HO-1: TBI vs. sham, ***p<0.001; PSD95: TBI vs. sham, ***p<0.00 1; Syn: TBI vs. sham, ***p<0.001, two-tailed t-test. All data were represented as mean ± SEM.

**
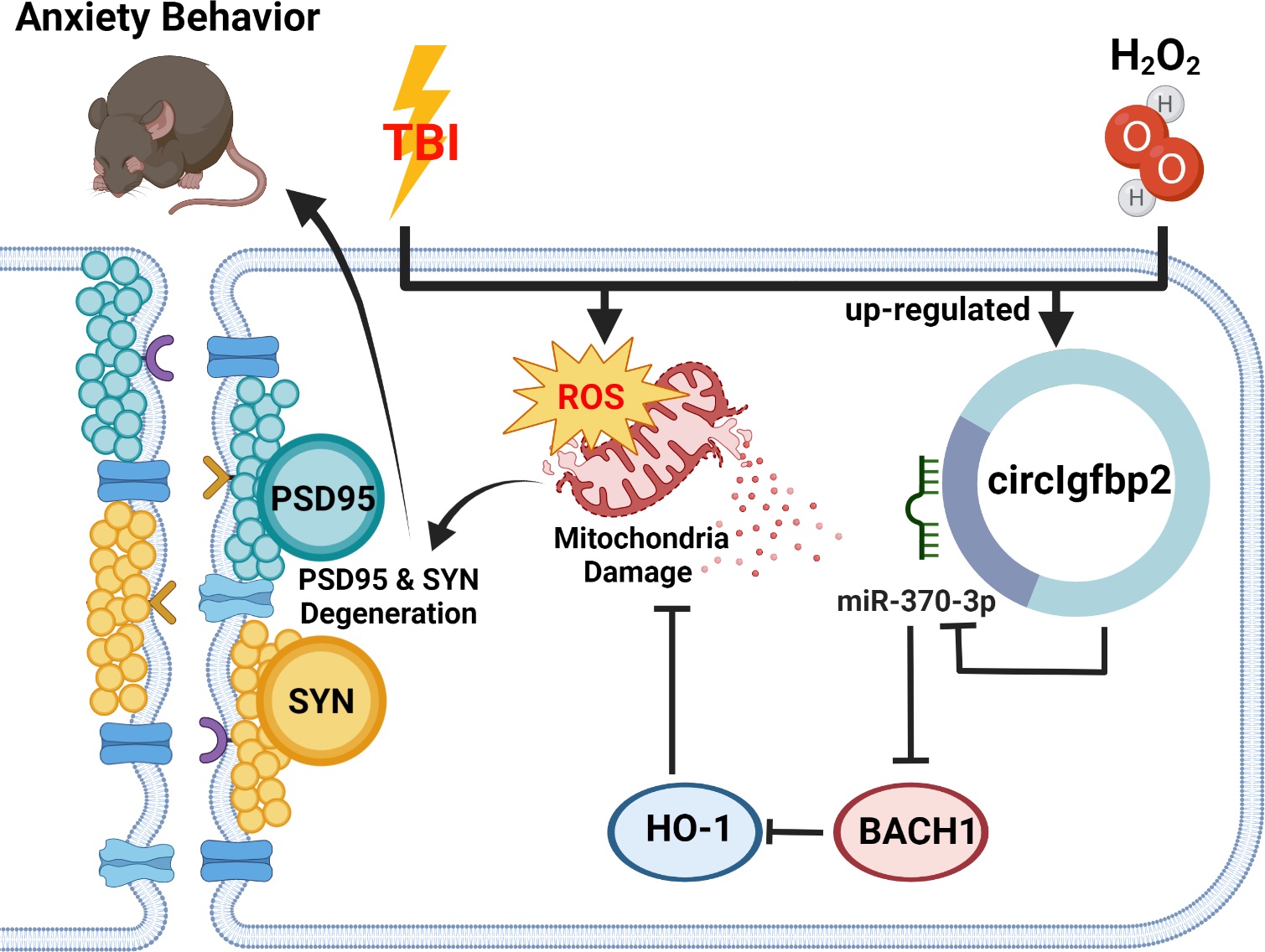
**

**Supplementary Fig. 3 The schematic model shows that circigfbp2 aggravates mitochondrial oxidative stress and leads to neurological dysfunction.** TBI or H_2_O_2_ intervention results in mitochondrial oxidative stress and upregulation of circIgfbp2. The upregulated circIgfbp2 adsorbs miR-370-3p as a sponge, which elevates the expression of BACH1. The upregulated BACH1 then leads to the decrease of HO-1 expression, which aggravates mitochondrial damage. Mitochondrial oxidative stress in neuronal cells also leads to the degradation of PSD95 and Syn, which impairs neural function. CircIgfbp2 can be used as a new target for anxiety and sleep disorders after TBI.
