# Supplementary figures and images for: A novel circular RNA, circIgfbp2, links neural plasticity and anxiety through targeting mitochondrial dysfunction and oxidative stress-induced synapse dysfunction after traumatic brain injury
Source: Mol Psychiatry. 2022 Aug 2;27(11):4575–89. doi: 10.1038/s41380-022-01711-7 (PMC9734054; doi:10.1038/s41380-022-01711-7)

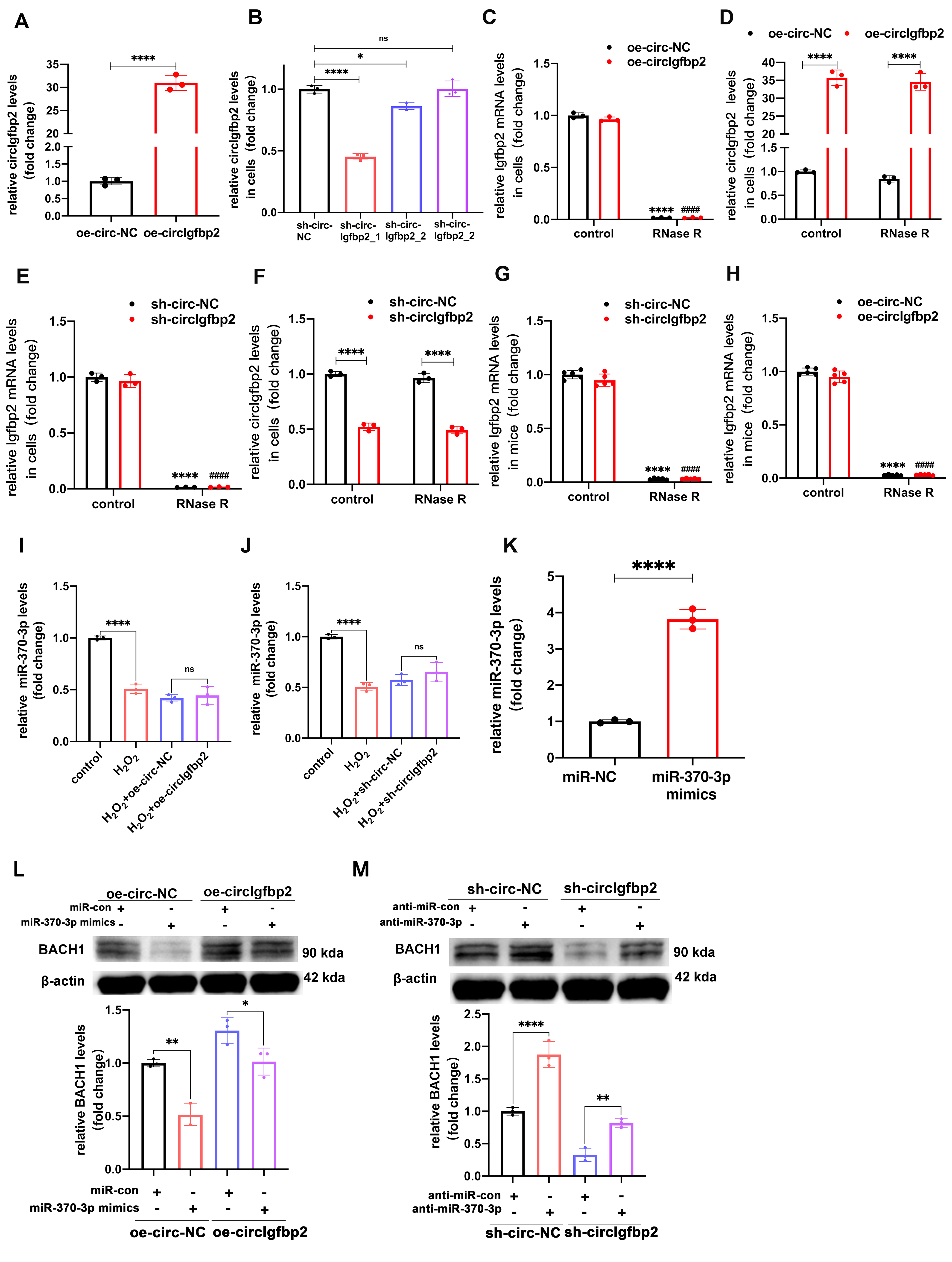

Supplement: Supplementary file 6 — Supplementary Fig. 1 [file 41380_2022_1711_MOESM6_ESM.tif]

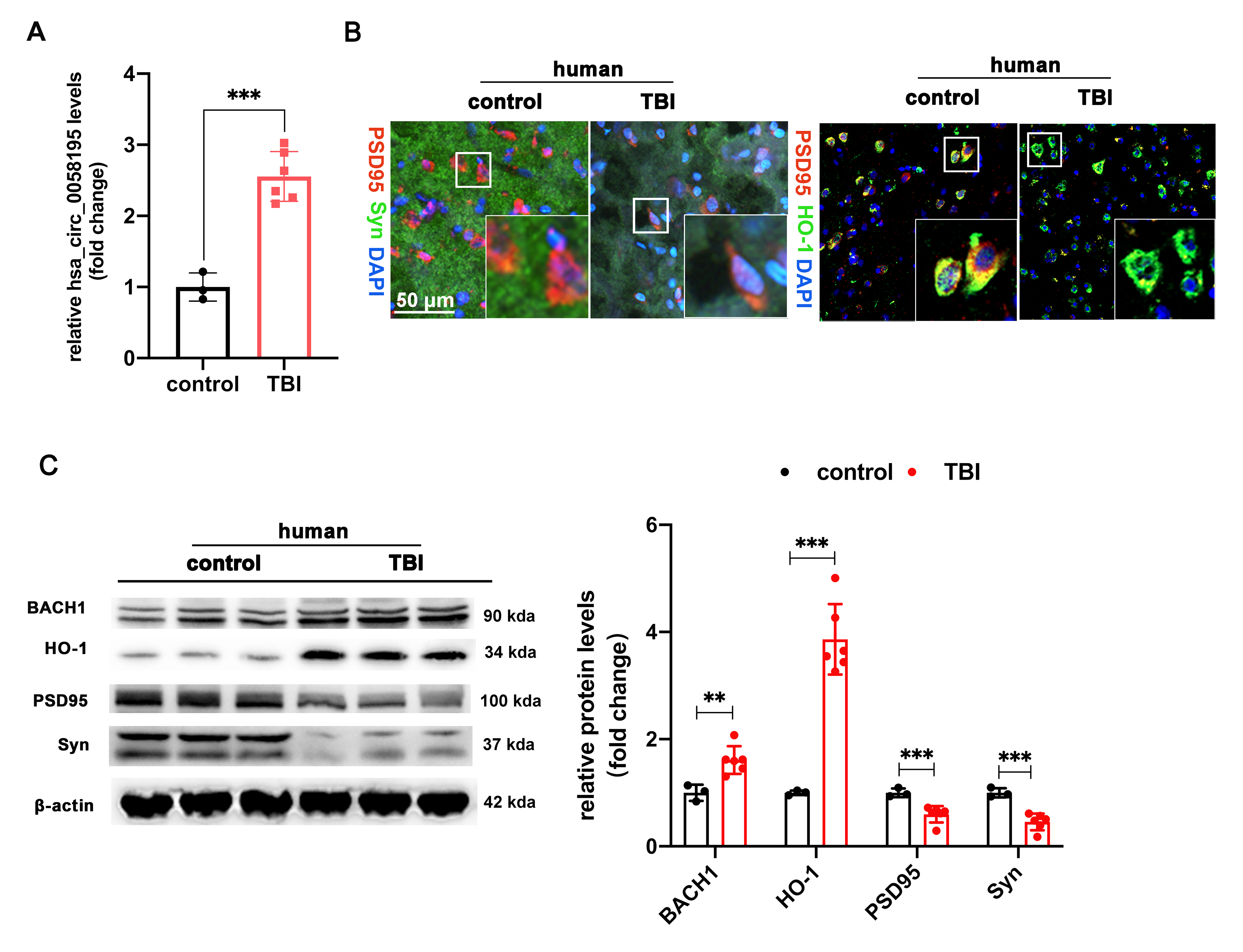

Supplement: Supplementary file 7 — Supplementary Fig. 2 [file 41380_2022_1711_MOESM7_ESM.tif]

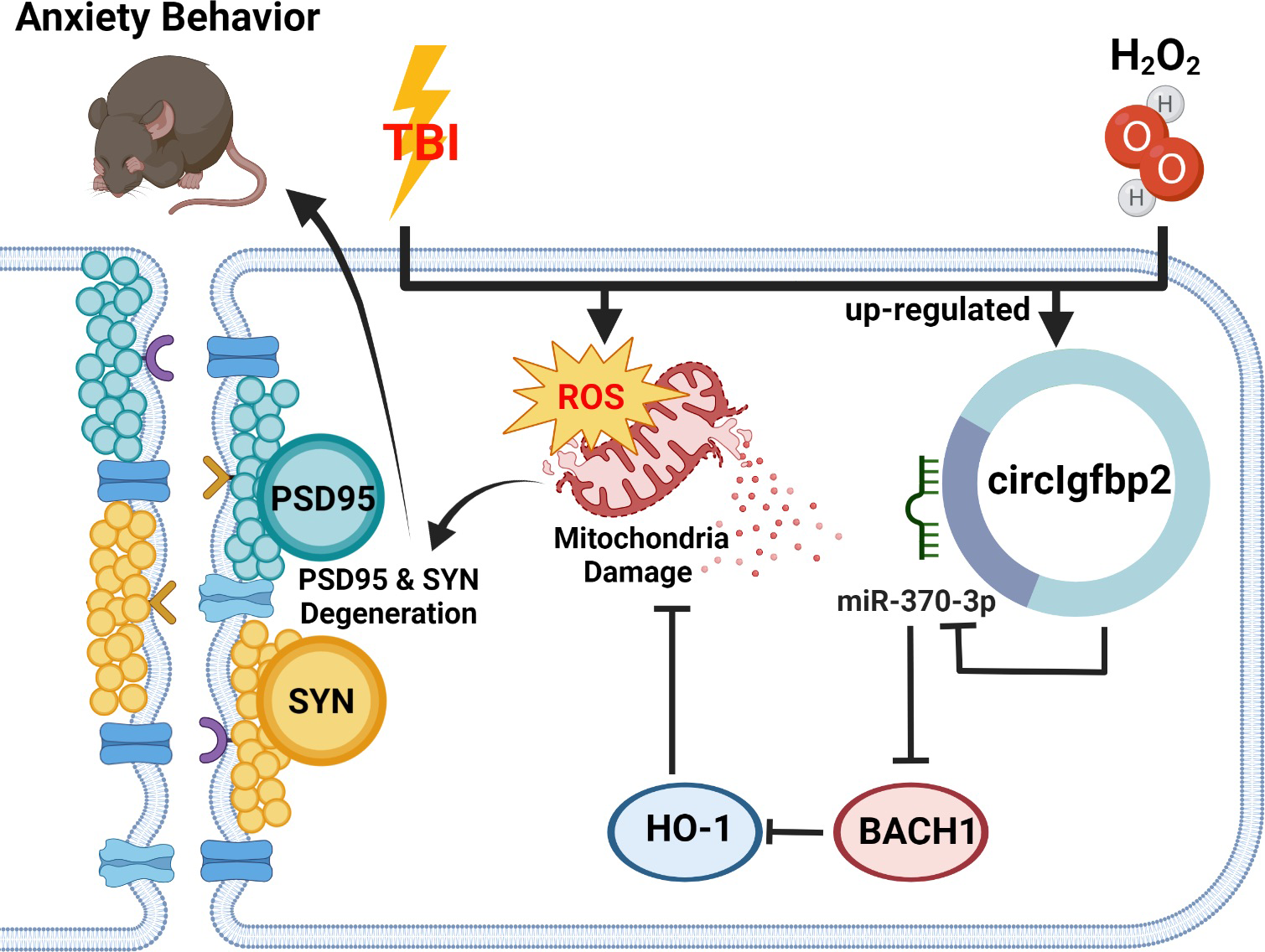

Supplement: Supplementary file 8 — Supplementary Fig. 3 [file 41380_2022_1711_MOESM8_ESM.tif]
